# LAG3 in gastric cancer: it’s complicated

**DOI:** 10.1007/s00432-023-04954-1

**Published:** 2023-06-14

**Authors:** Dita Ulase, Hans-Michael Behrens, Sandra Krüger, Steffen M. Heckl, Ulrike Ebert, Thomas Becker, Christoph Röcken

**Affiliations:** 1 Department of Pathology, University Hospital Schleswig-Holstein, Campus Kiel, Arnold-Heller-Str. 3, Building U33, 24105 Kiel, Germany; 2 Department of Internal Medicine II, University Hospital Schleswig-Holstein, Campus Kiel, Arnold-Heller-Str. 3, 24105 Kiel, Germany; 3 Department of General Surgery, Visceral, Thoracic, Transplantation and Pediatric Surgery, University Hospital Schleswig-Holstein, Campus Kiel, Arnold-Heller-Str. 3, 24105 Kiel, Germany

**Keywords:** Gastric cancer, Lymphocyte activation gene 3 protein, Prognosis, Tumor-infiltrating lymphocytes, Tumor microenvironment

## Abstract

**Purpose:**

Lymphocyte activation gene 3 (LAG3) is thought to contribute to T cell exhaustion within the tumor microenvironment of solid tumors. This study aimed to analyze the spatial distribution of LAG3 + cells in relation to clinicopathological and survival data in a large set of 580 primary resected and neoadjuvantly treated gastric cancers (GC).

**Methods:**

LAG3 expression was evaluated in tumor center and invasive margin using immunohistochemistry and whole-slide digital image analysis. Cases were divided into LAG3-low and LAG3-high expression groups based on (1) median LAG3 + cell density, (2) cut-off values adapted to cancer-specific survival using *Cutoff Finder* application.

**Results:**

Significant differences in spatial distribution of LAG3 + cells were observed in primarily resected GC, but not in neoadjuvantly treated GC. LAG3 + cell density showed evident prognostic value at following cut-offs: in primarily resected GC, 21.45 cells/mm^2^ in tumor center (17.9 vs. 10.1 months, *p* = 0.008) and 208.50 cells/mm^2^ in invasive margin (33.8 vs. 14.7 months, *p* = 0.006); and in neoadjuvantly treated GC, 12.62 cells/mm^2^ (27.3 vs. 13.2 months, *p* = 0.003) and 123.00 cells/mm^2^ (28.0 vs. 22.4 months, *p* = 0.136), respectively. Significant associations were found between LAG3 + cell distribution patterns and various clinicopathological factors in both cohorts. In neoadjuvantly treated GC, LAG3 + immune cell density was found to be an independent prognostic factor of survival (HR = 0.312, 95% CI 0.162–0.599, *p* < 0.001).

**Conclusion:**

In this study, a higher density of LAG3 + cells was associated with favorable prognosis. Current results support the need for extended analysis of LAG3. Differences in the distribution of LAG3 + cells should be considered, as they could influence clinical outcomes and treatment responses.

**Graphical abstract:**

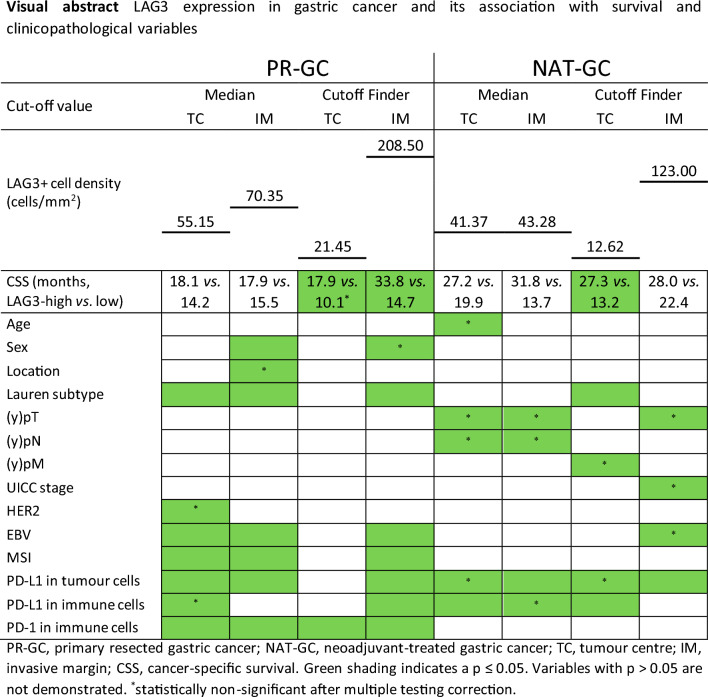

**Supplementary Information:**

The online version contains supplementary material available at 10.1007/s00432-023-04954-1.

## Introduction

Adenocarcinoma of the stomach and gastroesophageal junction (GC) is a heterogeneous disease that shows striking variations in epidemiology, etiology, risk factors, pathogenesis, topography, histological features, and prognosis. It remains the 5th most common and 4th deadliest cancer worldwide (Sung et al. 2021). Treatment options are still limited because of advanced-stage diagnoses that are common in the Western world (Arnold et al. 2019; National Cancer Institute).

Tumor immunotherapy has become one of the major therapeutic strategies in oncology (Galluzzi et al. 2014; van den Bulk et al. 2018; He and Xu 2020). Prominent of these therapies are immune checkpoint inhibitors (ICI) that target immune checkpoints, inhibitory or stimulatory proteins in immune cells, and/or tumor cells that modulate immune responses. Furthermore, immune checkpoints are involved in many processes of tumor cell metabolism and are related to epithelial–mesenchymal transition, metastasis, drug resistance, anti-apoptosis, and angiogenesis (Zhang and Zheng 2020). ICI block the transmission of inhibitory signals, stimulate the activation of cytotoxic T lymphocytes (CTLs), and boost the anti-tumor response of T lymphocytes (Shan et al. 2020). Current approved ICI are directed against programmed cell death 1 and its ligand (PD-1/PD-L1) and cytotoxic T-lymphocyte-associated protein 4 (CTLA4). However, many patients do not respond to ICI or the response is limited because of innate or acquired resistance (Shergold et al. 2019). Targeting other co-inhibitory receptors within the tumor microenvironment (TME), including simultaneous inhibition of multiple immune checkpoints, may provide new opportunities for immunotherapy.

Lymphocyte activation gene 3 (LAG3), also known as CD223, is a type I transmembrane protein expressed on a variety of immune cell types including activated CD4 + and CD8 + T cells, natural killer (NK) cells, NKT cells, and regulatory T cells (Triebel et al. 1990; Huang et al. 2004). Like other inhibitory receptors, it is essential to control T cell activation and to prevent autoimmunity (Andrews et al. 2017). LAG3 structurally resembles CD4 co-receptor and can interact with MHC class II molecules (Baixeras et al. 1992). Similar to *CD4*, the *LAG3* gene is located on chromosome 12. Although both molecules are closely related (Triebel et al. 1990), they exhibit different functions due to dissimilarities in their cytoplasmic domains (Workman et al. 2002; Andrews et al. 2017). Unlike CD4 and any other known immune checkpoints, the LAG3 cytoplasmic tail contains specific KIEELE, FxxL, and EP motifs, which are thought to be essential for its inhibitory function (Anderson et al. 2016; Maeda et al. 2019).

Despite over 30 years of research involving LAG3, the exact mechanism by which LAG3 and its binding partner(s) contribute to T cell suppression is not completely understood. LAG3 is strongly upregulated upon continuous T cell stimulation due to persistent exposure to tumor antigens (Andrews et al. 2017). This inhibitory signal is thought to contribute to T cell exhaustion. Exhausted T cells lose their ability to proliferate and to perform effector functions including cytokine production and degranulation (Wherry and Kurachi 2015). Besides its well-recognized ligand MHC class II, several alternate LAG3 ligands have been reported, including liver sinusoidal endothelial cell lectin (Xu et al. 2014), galectin-3 (Kouo et al. 2015), and fibrinogen-like protein 1 (Wang et al. 2019). Interestingly, a recent study demonstrated that LAG3 could inhibit T cell receptor (TCR) signaling in the absence of binding MHC II class (Guy et al. 2022).

In vitro studies have shown that LAG3 expression level strongly correlates with its inhibitory function, and changes in the amount of LAG3 on the cell surface directly affect its inhibitory effect (Maeda et al. 2019). LAG3 is co-expressed with other inhibitory receptors like PD-1, CTLA4, T-cell immunoglobulin and mucin domains-containing protein 3 (TIM3), and T cell immuno-receptor with Ig and ITIM domains (TIGIT) (Blank et al. 2019). It has been suggested that the higher the number of co-expressed inhibitory receptors, the more severe is the T cell exhaustion (Wherry and Kurachi 2015). Dual blockade of PD-1 and LAG3 has led to decreased tumor growth and enhanced anti-tumor immunity in mouse models (Woo et al. 2012). In patients with previously untreated metastatic or un-resectable melanoma, combination of relatlimab (anti-LAG3 antibody) and nivolumab (anti-PD-1 antibody) has increased the median progression-free survival more than two times when compared to PD-1 inhibition alone (Tawbi et al. 2022). Recently, the first such combination immunotherapy has been approved by the U.S. Food and Drug Administration (Paik 2022).

The effectiveness of LAG3-targeted therapies in GC has been evaluated in several clinical trials (https://clinicaltrials.gov/, accessed on 10.04.2023). However, the clinicopathological significance and prognostic role of LAG3 protein expression in GC remain unclear, showing discrepant results with regard to patient prognosis (Lee et al. 2019; Park et al. 2021; Lv et al. 2021). To our knowledge, there are no published data on LAG3 expression in GC of Western origin. Based on previous studies, we hypothesize that: (1) LAG3 expression varies among histological and molecular subtypes of GC, and (2) high density of LAG3 + cells is related to advanced clinicopathological features and poor outcome in GC. To check our hypotheses and to deepen our understanding of the role of LAG3 in GC, we investigated the clinicopathological and prognostic significance of LAG3 in a large cohort of primarily resected chemotherapy-naïve GC (PR-GC). Additionally, the need for information on the expression patterns of immune checkpoints in tumors after neoadjuvant therapy is increasing. Therefore, we evaluated LAG3 expression also in a set of neoadjuvantly treated GC (NAT-GC).

## Methods

### Study population

A well-characterized cohort of patients with GC was included in this study. Formalin-fixed and paraffin-embedded tissue samples from the primary tumor site were analyzed. Patients in the PR-GC sub-cohort underwent primary total gastrectomy or surgical resection between 1997 and 2009 at the University Hospital Schleswig–Holstein, Kiel, Germany. Patients in the NAT-GC sub-cohort received preoperative chemo-radiation or perioperative chemotherapy and underwent total gastrectomy or surgical resection between 1998 and 2019. The following exclusion criteria were used: (a) diagnosis other than adenocarcinoma and (b) insufficient tissue for tumor compartment analysis, that is, no residual tumor in the NAT-GC. Clinicopathological characteristics were collected from previous records, including sex, age at diagnosis, tumor localization, type by Laurén, pathologic (pTNM) or post-neoadjuvant (ypTNM) stage according to the 8th edition of the UICC guidelines (Brierley et al. 2017), status of resection lines (pR), and lymphatic or venous invasion (LVI). For survival analysis, the date of surgery and date of death or last follow-up were used. The *Helicobacter pylori*, microsatellite instability (MSI)-, Epstein–Barr virus (EBV)-, HER2-, MET-, and PD-1/PD-L1-status was available from previous studies of the cohort (Warneke et al. 2013; Metzger et al. 2016; Böger et al. 2016, 2017; Mathiak et al. 2017; Schoop et al. 2020).

### Immunohistochemical detection of LAG3

Immunohistochemical staining was performed on whole tissue sections using the Bondmax automated system (Leica Biosystems, Wetzlar, Germany). The LAG3 monoclonal antibody (Clone D2G4O, Cell Signaling, Leiden, the Netherlands) was used at a 1:50 dilution. Slides were pre-treated with ER2 (Leica Biosystems) for 20 min, and visualization was performed using the Bond Polymer Refine Detection Kit (Leica Biosystems).

### Evaluation of LAG3 immunostaining

**Fig. 1:**
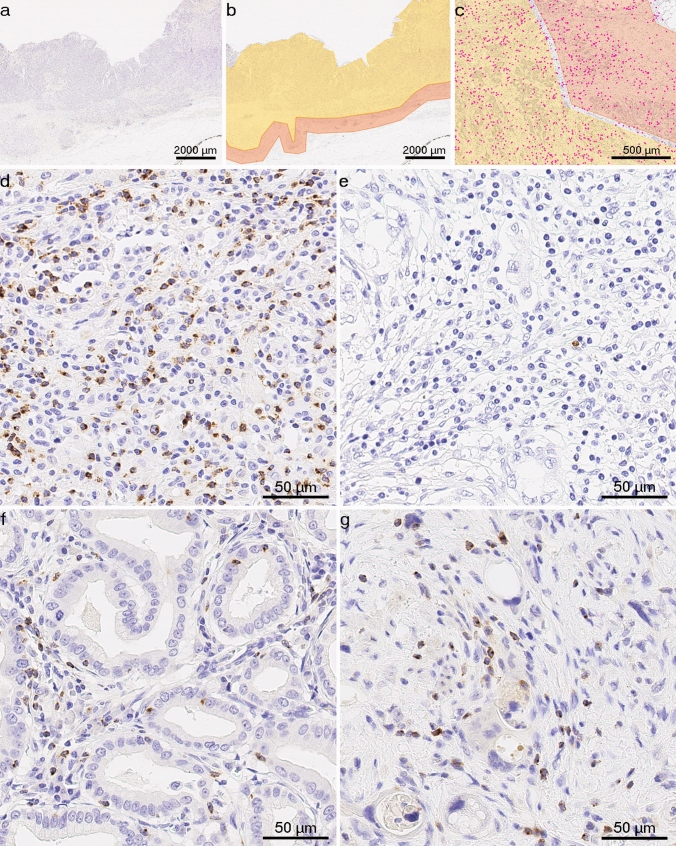
LAG3 + immune cells in gastric cancer and cancer of gastroesophageal junction: **a**–**c**, representative images from the viewer and painting program *VMP*, which was used to mark the tumor center (yellow) and invasive margin (orange), magenta dots **c** indicate LAG3 + cells identified using *Definiens Tissue Studio*. Anti-LAG3 immunostaining, original magnification × 12.5 (**a**, **b**) and × 50 (**c**); **d**–**g**, representative tissue sections with LAG3 + tumor-infiltrating immune cells in primary resected and neoadjuvantly treated tumors: **d** high expression in EBV-associated gastric adenocarcinoma; **e** low expression in unclassified gastric carcinoma with prominent tumor-associated inflammatory reaction in the stroma; **f** LAG3 + immune cells were present in both the stromal and intraepithelial compartments of this gastric intestinal-type adenocarcinoma; **g** LAG3 + immune cells in gastric cancer treated with neoadjuvant therapy. Anti-LAG3 immunostaining, original magnification × 400

Digital images of whole tissue sections were obtained using a Leica SCN400 scanner (Leica Biosystems, Nussloch, Germany) at 40 × nominal magnification, corresponding to a resolution of 0.25 µm per pixel. To detect LAG3 + cells, image analysis was performed using Definiens Tissue Studio (version 4.4.3, Definiens, Munich, Germany). A machine learning algorithm using Definiens Composer Technology was trained with four representative images to recognize LAG3 + cells. To avoid false results (e.g., due to large areas of necrotic debris, surface ulcerations, mucin pools, folded tissue, or holes), marking of distinct tumor compartments was carried out manually using the viewer and painting program VMP (Fig. 1a–c). The invasive margin (IM) was defined as a narrow band-like area with a width of up to 1000 µm centered on the border separating cancer cells from the host tissue (Hendry et al. 2017). The tumor center (TC) was defined as the remaining tumor area comprising cancer cells and desmoplastic stroma. In NAT-GC, the tumor center could include surrounding scarred tissue that had reacted to NAT. LAG3 + cell densities were individually computed for TC and IM.

### Statistical analysis

Data were analyzed using SPSS 25.0.0.2 (IBM Corporation, New York, USA). A significance level of *p* < 0.05 was assumed. Overall survival (OS) and cancer-specific survival (CSS) were defined as the time from the date of surgery until death due to any cause and death due to GC, respectively. The median LAG3 + cell density was calculated separately for each cohort. Because of differences in median densities between both cohorts, as well as tumor compartments, it was decided to analyze them separately and use individual cut-offs. To incorporate LAG3 + immune cell density in further statistical analyses, it was dichotomized into low and high LAG3 expression groups: (1) by median number; (2) using *Cutoff Finder* web application (Budczies et al. 2012), in which the optimal cut-off values were generated based on CSS. Survival curves were estimated using the Kaplan–Meier method and compared using the log-rank test. Multivariate survival analysis was performed using backward stepwise (likelihood ratio) Cox regression models and included all variables with *p* < 0.100 in univariate survival analysis. Associations with demographic and clinicopathological variables were analyzed using cross-tabulation analysis and Kendall’s tau test for ordinal variables or Fischer’s exact test for non-ordinal variables. To account for the false discovery rate, the Benjamini–Hochberg (Simes) method was applied to the pool of all *p* values of this study (*n* = 172) (Benjamini and Hochberg 1995). All the *p* values are given uncorrected. Those *p* values which have lost significance are marked accordingly.

## Results

### Cohort characteristics

A total of 580 patients (441 with PR-GC and 139 with NAT-GC) were included in this study. For further information on baseline characteristics, see Supplementary Table 1. The median OS for the PR-GC cohort was 14.7 months [95% confidence interval (CI) 12.6–16.7 months], and the median CSS was 16.0 months (95% CI 13.5–18.5). In NAT-GC, the median OS was 22.4 months (95% CI 17.3–27.4), and CSS was 24.6 months (95% CI 19.9–29.4).

### Accumulation of LAG3 + cells in GC

LAG3 + immune cells were detected in both the stromal and intraepithelial compartments. The staining pattern varied from weak and dot-like to strong and membranous/cytoplasmic (Fig. 1d–g), which was also recently demonstrated in melanoma (Johnson et al. 2022). The distribution of LAG3 + cell densities differed significantly between the two sub-cohorts (*p* < 0.0001). In PR-GC, the median density of LAG3 + immune cells was 55.15 cells/mm^2^ (range 3.57–1687.63) in TC and 70.35 cells/mm^2^ (1.91–1858.35) in IM, and it differed significantly between both compartments (*p* < 0.001). This pattern of distribution was not observed in the NAT-GC, where the median numbers were 41.37 (3.56–543.43) in the TC and 43.28 (3.60–537.73) in the IM.

### Survival analysis

To incorporate LAG3 expression in the survival analysis, median LAG3 + cell densities were used in the first step to dichotomize cohorts into low and high LAG3 expression groups. In PR-GC, no significant differences were found in OS (data not shown) and CSS between the two groups (Suppl. Table 2; Suppl. Fig. 1).

Similar results were found in the NAT-GC sub-cohort when comparing low *vs*. high LAG3 expression in TC (Suppl. Table 2; Suppl. Fig. 2). Interestingly, NAT-GC patients with higher LAG3 + cell density at the IM had significantly longer OS than in the LAG3 low subgroup (28.0 vs. 13.3 months, *p* = 0.025, log-rank test; data not shown).

### LAG3 expression predicts favorable prognosis when divided by biological cut-off

**Fig. 2:**
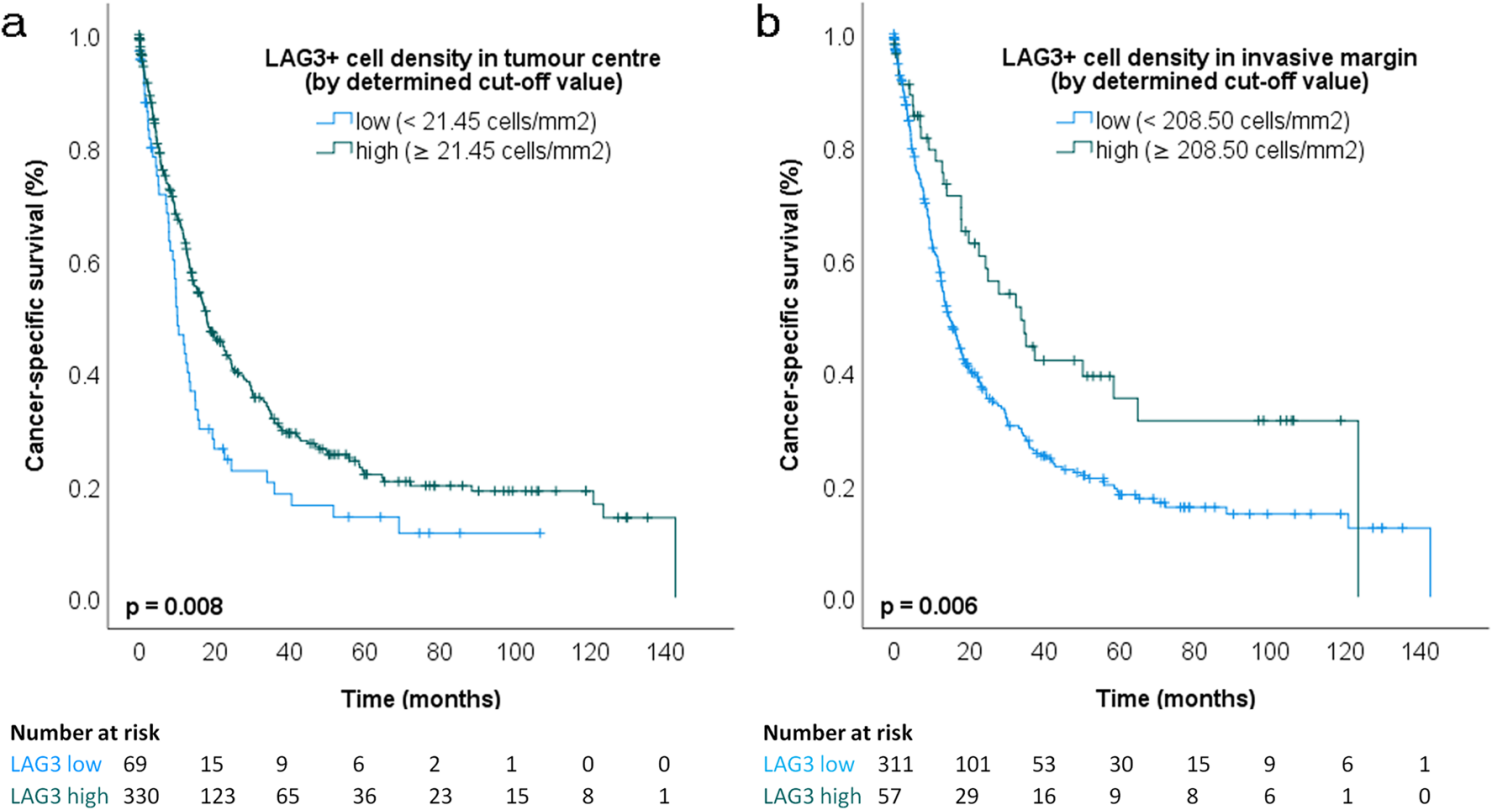
Kaplan–Meier curves in primary resected gastric cancer: cancer-specific survival according to LAG3 + cell density groups split by determined cut-off values (**a** tumor center, *p* = 0.008, log-rank test; **b** invasive margin, *p* = 0.006, log-rank test; small vertical lines in the graph indicate censored data)

Dichotomization of patient cohorts at median values may not reflect biological relevant cut-offs, as it was previously shown by our group, e.g., for neutrophil counts in GC (Clausen et al. 2020). Therefore, we explored an alternative dichotomization approach for LAG3 using *Cutoff Finder* (Budczies et al. 2012) and CSS as outcome measure. *Cutoff Finder* is a web application, which enables detection of optimal biomarker cut-offs. Interestingly, using this tool in PR-GC, the most significant cut-offs were 21.45 cells/mm^2^ in TC and 208.50 cells/mm^2^ at IM. Now, the median CSS of patients with high LAG3 expression in TC was 17.9 months (95% CI 13.7–22.1 months) in comparison to 10.1 months (95% CI 7.5–12.7) of LAG3-low patients (*p* = 0.008). After splitting cases with high and low LAG3 expression in the IM, the median CSS reached 33.8 months (95% CI 21.4–46.3) vs. 14.7 months (95% CI 12.1–17.2), respectively (*p* = 0.006) (Fig. 2; Suppl. Table 2). However, when using multivariate analysis, LAG3 expression was not found to be an independent prognostic factor (Table 1).

**Table 1:** Multivariate analysis: Independent predictors for cancer-specific survival using a Cox proportional hazards model; variables with *p* < 0.100 by univariate analysis were included in the multivariate analysis

Variable	Primarily resected GC			Neoadjuvantly treated GC		
	HR	95% CI	*p* value	HR	95% CI	*p* value
Laurén phenotype			NS	Not included		
UICC stage			< 0.001			0.034
II A/B vs. I A/B	2.544	1.330–4.866	0.005	2.014	0.625–6.492	NS
III A/B/C vs. I A/B	5.901	3.214–10.833	< 0.001	3.715	1.285–10.738	0.015
IV vs. I A/B	8.180	4.156–16.100	< 0.001	4.821	1.448–16.052	0.010
R status (R1/2 vs. R0)	2.891	1.957–4.271	< 0.001	2.775	1.361–5.660	0.005
Lympho-vascular invasion (L1 vs. L0)			NS			NS
Venous invasion (V1 vs. V0)			NS	4.690	2.089–10.528	< 0.001
MET status (positive vs. negative)	2.009	1.171–3.447	0.011			NS
MSI status (MSI vs. MSS)			NS	Not included		
PD-L1 in tumor cells (positive vs. negative)			NS	Not included		
PD-L1 in immune cells (positive vs. negative)	0.643	0.478–0.867	0.004	Not included		
PD-1 in immune cells			NS	Not included		
LAG3 + cell density in TC (high vs. low)^a^			NS	0.312	0.162–0.599	< 0.001
LAG3 + cell density at IM (high vs. low)^a^			NS	Not included		

*GC* gastric cancer, *HR* hazard ratio, *CI* confidence interval, *UICC* Union for International Cancer Control, *TC* tumor center, *IM* invasive margin, *NS* not statistically significant

^a^Based on the adjusted cut-off value of Cutoff Finder

**Fig. 3:**
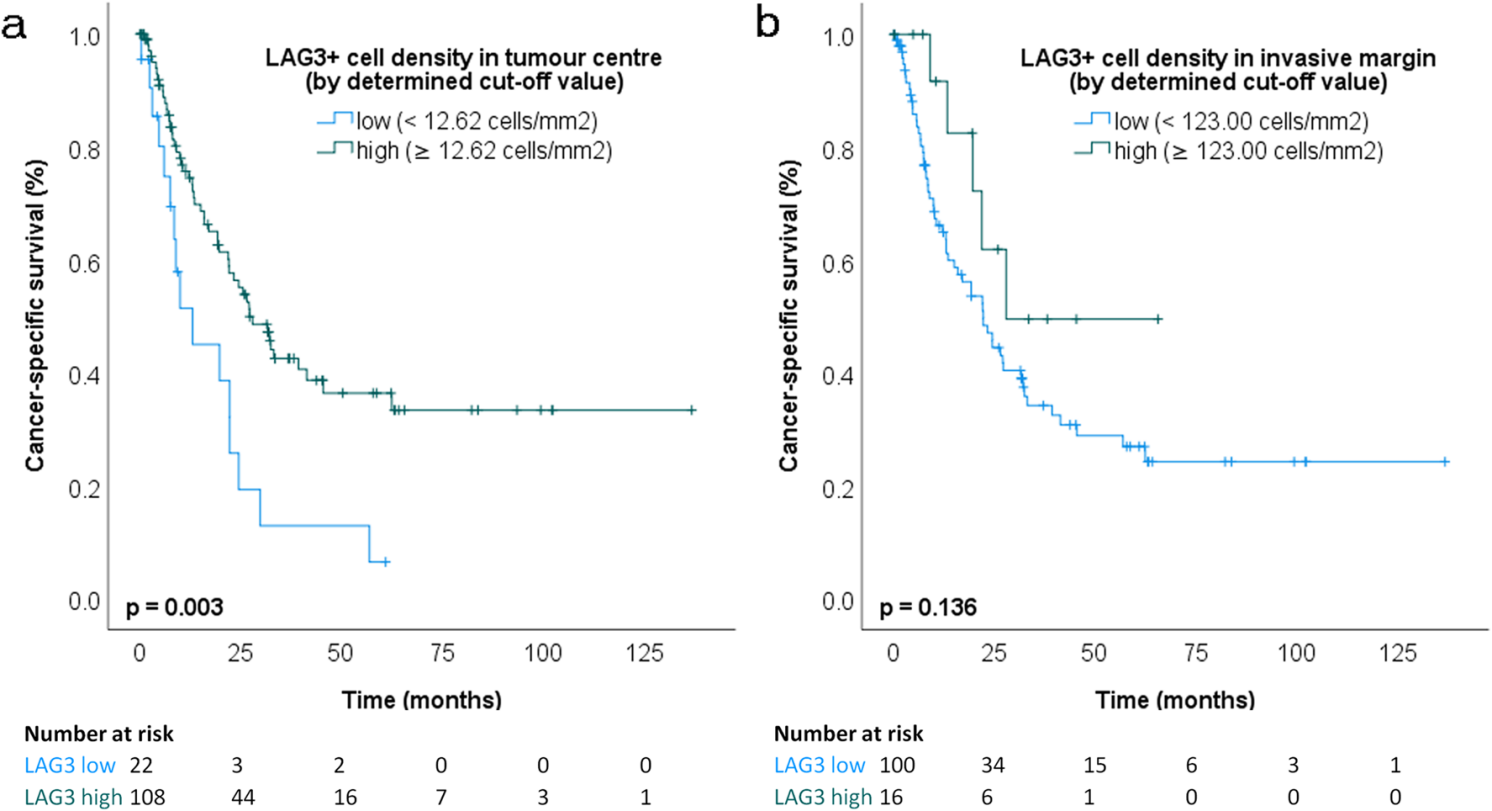
Kaplan–Meier curves in neoadjuvantly treated gastric cancer: cancer-specific survival according to LAG3 + cell density groups split by determined cut-off values (**a** tumor center, *p* = 0.003, log-rank test; **b** invasive margin, *p* = 0.136, log-rank test; small vertical lines in the graph indicate censored data)

In NAT-GC, the most significant cut-off of LAG3 + cell density in TC was 12.62 cells/mm^2^. The median CSS was 27.3 months (95% CI 19.6–35.1) and 13.2 months (95% CI 5.6–20.8) in patients with high *vs.* low LAG3 expression, respectively (*p* = 0.003). LAG3 expression in TC was an independent prognosticator of CSS (Table 1). There was no significant difference in median CSS among LAG3 groups of IM even at the most optimal calculated cut-off (123.00 cells/mm^2^): 28.0 months (95% CI non-calculable), LAG3-high, vs. 22.4 months (15.5–29.2), LAG3-low (*p* = 0.136) (Fig. 3; Suppl. Table 2). These data show that the biological effect of LAG3 depends on cell numbers, location, and treatment.

### Association with demographical and clinicopathological features

The associations between LAG3 expression (dichotomized by biological cut-offs) and clinicopathological characteristics are summarized in Tables 2 and 3. In PR-GC (Table 2), a significant association was found between LAG3 expression in IM and the Lauren phenotype (*p* < 0.001). Enrichment of LAG3 + cells in the IM was more likely to be observed in males (*p* = 0.050). Higher LAG3 + density in both compartments was associated with PD-1 expression (*p* = 0.004, TC, and *p* = 0.002, IM). LAG3 + cell density in IM showed a correlation with EBV, MSI- and PD-L1-status (*p* < 0.001). No significant associations were found between LAG3 expression and patient age, tumor location, pTNM categories, presence of LVI, pR status, *H. pylori* infection, and MET-status. For analysis based on median values, see Suppl. Table 3.

**Table 2:** Primarily resected GC: Association of LAG3 + cell density (dichotomized by determined cutoff) with demographical and clinicopathological patient characteristics

Characteristics	Tumor center							Invasive margin						
	Valid/missing		LAG3 low		LAG3 high		*p* value	Valid/missing		LAG3 low		LAG3 high		*p* value
	*N*	(%)	*n*	(%)	*n*	(%)		*n*	(%)	*n*	(%)	*n*	(%)	
Sex	441/0						0.515^a^	408/33						0.050^a,^*
Male	277	(62.8)	45	(16.2)	232	(83.8)		254	(62.3)	207	(81.5)	47	(18.5)	
Female	164	(37.2)	31	(18.9)	133	(81.1)		154	(37.7)	137	(89.0)	17	(11.0)	
Age	441/0						0.900^b^	408/33						0.276^b^
< 68 years	220	(49.9)	37	(16.8)	183	(83.2)		201	(49.3)	165	(82.1)	36	(17.9)	
≥ 68 years	221	(50.1)	39	(17.6)	182	(82.4)		207	(50.7)	179	(86.5)	28	(13.5)	
Location	438/3						1.000^a^	405/36						0.186^a^
Proximal stomach	141	(32.2)	24	(17.0)	117	(83.0)		130	(32.1)	105	(80.8)	25	(19.2)	
Distal stomach	297	(67.8)	51	(17.2)	246	(82.8)		275	(67.9)	237	(86.2)	38	(13.8)	
Laurén phenotype	441/0						0.678^a^	408/33						< 0.001^a^
Intestinal	226	(51.2)	39	(17.3)	187	(82.7)		216	(52.9)	177	(81.9)	39	(18.1)	
Diffuse	138	(31.3)	27	(19.6)	111	(80.4)		119	(29.2)	118	(99.2)	1	(0.8)	
Mixed	29	(6.6)	3	(10.3)	26	(89.7)		26	(6.4)	23	(88.5)	3	(11.5)	
Unclassified	48	(10.9)	7	(14.6)	41	(85.4)		47	(11.5)	26	(55.3)	21	(44.7)	
pT category	441/0						0.071^b^	408/33						0.167^b^
pT1 (a/b)	55	(12.5)	3	(5.5)	52	(94.5)		48	(11.8)	39	(81.3)	9	(18.8)	
pT2	48	(10.9)	7	(14.6)	41	(85.4)		47	(11.5)	36	(76.6)	11	(23.4)	
pT3	181	(41.0)	36	(19.9)	145	(80.1)		172	(42.2)	147	(85.5)	25	(14.5)	
pT4 (a/b)	157	(35.6)	30	(19.1)	127	(80.9)		141	(34.5)	122	(86.5)	19	(13.5)	
pT category	441/0						0.025^a,^*	408/33						0.109^a^
pT1 (a/b)/pT2	103	(23.4)	10	(9.7)	93	(90.3)		95	(23.3)	75	(78.9)	20	(21.1)	
pT3/pT4 (a/b)	338	(76.6)	66	(19.5)	272	(80.5)		313	(76.7)	269	(85.9)	44	(14.1)	
pN category	440/1						0.604^b^	408/33						0.283^b^
pN0	127	(28.9)	19	(15.0)	108	(85.0)		120	(29.4)	96	(80.0)	24	(20.0)	
pN1	60	(13.6)	13	(21.7)	47	(78.3)		57	(14.0)	49	(86.0)	8	(14.0)	
pN2	77	(17.5)	12	(15.6)	65	(84.4)		69	(16.9)	61	(88.4)	8	(11.6)	
pN3 (a/b)	176	(40.0)	32	(18.2)	144	(81.8)		162	(39.7)	138	(85.2)	24	(14.8)	
pN category	440/1						1.000^a^	408/33						0.136^a^
pN0	127	(28.9)	19	(15.0)	108	(85.0)		120	(29.4)	96	(80.0)	24	(20.0)	
pN +	313	(71.1)	57	(18.2)	256	(81.8)		288	(70.6)	248	(86.1)	40	(13.9)	
pM category	441/0						1.000^b^	408/33						0.286^b^
M0	358	(81.2)	62	(17.3)	296	(82.7)		335	(82.1)	279	(83.3)	56	(16.7)	
M1	83	(18.8)	14	(16.9)	69	(83.1)		73	(17.9)	65	(89.0)	8	(11.0)	
UICC stage	440/1						0.361^b^	408/33						0.197^b^
IA/IB	75	(17.0)	8	(10.7)	67	(89.3)		68	(16.7)	54	(79.4)	14	(20.6)	
IIA/IIB	96	(21.8)	18	(18.8)	78	(81.3)		93	(22.8)	79	(84.9)	14	(15.1)	
IIIA/IIIB/IIIC	186	(42.3)	36	(19.4)	150	(80.6)		174	(42.6)	146	(83.9)	28	(16.1)	
IV	83	(18.9)	14	(16.9)	69	(83.1)		73	(17.9)	65	(89.0)	8	(11.0)	
pR status	434/5						0.336^b^	403/38						0.673^b^
pR0	381	(87.4)	62	(16.3)	319	(83.7)		356	(88.3)	299	(84.0)	57	(16.0)	
pR1/pR2	55	(12.6)	12	(21.8)	43	(78.2)		47	(11.7)	41	(87.2)	6	(12.8)	
L category	421/20						0.239^b^	390/51						0.096^b^
L0	203	(48.2)	29	(14.3)	174	(85.7)		186	(47.7)	150	(80.6)	36	(19.4)	
L1	218	(51.8)	41	(18.8)	177	(81.2)		204	(52.3)	178	(87.3)	26	(12.7)	
V category	420/21						0.677^b^	389/52						0.512^b^
V0	373	(88.8)	61	(16.4)	312	(83.6)		345	(88.7)	292	(84.6)	53	(15.4)	
V1	47	(11.2)	9	(19.1)	38	(80.9)		44	(11.3)	35	(79.5)	9	(20.5)	
*H. pylori* status	374/67						0.563^a^	351/90						0.418^a^
Negative	317	(84.8)	51	(16.1)	266	(83.9)		295	(84.0)	247	(83.7)	48	(16.3)	
Positive	57	(15.2)	11	(19.3)	46	(80.7)		56	(16.0)	50	(89.3)	6	(10.7)	
HER2 status	412/29						0.482^a^	384/57						0.806^a^
Negative	378	(91.7)	69	(18.3)	309	(81.7)		350	(91.1)	295	(84.3)	55	(15.7)	
Positive	34	(8.3)	4	(11.8)	30	(88.2)		34	(8.9)	28	(82.4)	6	(17.6)	
MET status	430/11						0.238^a^	401/40						0.282^a^
Negative	397	(92.3)	71	(17.9)	326	(82.1)		374	(93.3)	312	(83.4)	62	(16.6)	
Positive	33	(7.7)	3	(9.1)	30	(90.9)		27	(6.7)	25	(92.6)	2	(7.4)	
EBV status	431/10						0.056^a^	400/41						< 0.001^a^
Negative	412	(95.6)	75	(18.2)	337	(81.8)		383	(95.8)	333	(86.9)	50	(13.1)	
Positive	19	(4.4)	0	(0)	19	(100)		17	(4.2)	4	(23.5)	13	(76.5)	
MSI status	429/12						0.331^a^	398/43						< 0.001^a^
Negative (MSS)	397	(92.5)	72	(18.1)	325	(81.9)		366	(92.0)	316	(86.3)	50	(13.7)	
Positive	32	(7.5)	3	(9.4)	29	(90.6)		32	(8.0)	18	(56.3)	14	(43.8)	
PD-L1 in tumor cells^c^	419/22						0.227^b^	388/53						< 0.001^b^
Negative (IRS ≤ 2)	319	(76.1)	59	(18.5)	260	(81.5)		292	(75.3)	266	(91.1)	26	(8.9)	
Positive (IRS > 2)	100	(23.9)	13	(13.0)	87	(87.0)		96	(24.7)	58	(60.4)	38	(39.6)	
PD-L1 in immune cells^c^	419/22						0.593^b^	388/53						< 0.001^b^
Negative (QS ≤ 1)	267	(63.7)	48	(18.0)	219	(82.0)		243	(62.6)	219	(90.1)	24	(9.9)	
Positive (QS > 1)	152	(36.3)	24	(15.8)	128	(84.2)		145	(37.4)	105	(72.4)	40	(27.6)	
PD-1 in immune cells	422/19						0.004^a^	391/50						0.002^a^
Not present	191	(45.3)	44	(23.0)	147	(77.0)		174	(44.5)	157	(90.2)	17	(9.8)	
Present	231	(54.7)	28	(12.1)	203	(87.9)		217	(55.5)	170	(78.3)	47	(21.7)	

*IRS* immunoreactivity score, *QS* quantity score

*Statistically non-significant after multiple testing correction

^a^Fisher's exact test

^b^Kendall's tau test

^c^Cutoffs used by Böger et al.

**Table 3:** Neoadjuvantly treated GC: Association of LAG3 + cell density (dichotomized by determined cutoff) with demographical and clinicopathological patient characteristics

Characteristics	Tumor center							Invasive margin						
	Valid/missing		LAG3 low		LAG3 high		*p* value	Valid/missing		LAG3 low		LAG3 high		*p* value
	*n*	(%)	*n*	(%)	*n*	(%)		*n*	(%)	*n*	(%)	*n*	(%)	
Sex	139/0						0.404^a^	124/15						0.350^a^
Male	110	(79.1)	16	(14.5)	94	(85.5)		98	(79.0)	86	(87.8)	12	(12.2)	
Female	29	(20.9)	6	(20.7)	23	(79.3)		26	(21.0)	21	(80.8)	5	(19.2)	
Age	139/0						0.170^b^	124/15						0.124^b^
< 64 years	69	(49.6)	14	(20.3)	55	(79.7)		59	(47.6)	54	(91.5)	5	(8.5)	
≥ 64 years	70	(50.4)	8	(11.4)	62	(88.6)		65	(52.4)	53	(81.5)	12	(18.5)	
Location	139/0						0.074^a^	124/15						0.571^a^
Proximal stomach	99	(71.2)	12	(12.1)	87	(87.9)		88	(71.0)	77	(87.5)	11	(12.5)	
Distal stomach	40	(28.8)	10	(25.0)	30	(75.0)		36	(29.0)	30	(83.3)	6	(16.7)	
Laurén phenotype	139/0						< 0.001^a^	124/15						0.068^a^
Intestinal	73	(52.5)	4	(5.5)	69	(94.5)		68	(54.8)	58	(85.3)	10	(14.7)	
Diffuse	27	(19.4)	11	(40.7)	16	(59.3)		23	(18.5)	23	(100)	0	(0.0)	
Mixed	29	(20.9)	5	(17.2)	24	(82.8)		24	(19.4)	18	(75.0)	6	(25.0)	
Unclassified	10	(7.2)	2	(20.0)	8	(80.0)		9	(7.3)	8	(88.9)	1	(11.1)	
ypT category	139/0						0.333^b^	124/15						0.013^b,^*
pT1 (a/b)	18	(13.0)	3	(16.7)	15	(83.3)		14	(11.3)	11	(78.6)	3	(21.4)	
pT2	23	(16.5)	3	(13.0)	20	(87.0)		20	(16.1)	14	(70.0)	6	(30.0)	
pT3	88	(63.3)	12	(13.6)	76	(86.4)		81	(65.3)	73	(90.1)	8	(9.9)	
pT4 (a/b)	10	(7.2)	4	(40.0)	6	(60.0)		9	(7.3)	9	(100)	0	(0.0)	
ypT category	139/0						1.000^a^	124/15						0.018^a,^*
pT1 (a/b)/pT2	41	(29.5)	6	(14.6)	35	(85.4)		34	(27.4)	25	(73.5)	9	(26.5)	
pT3/pT4 (a/b)	98	(70.5)	16	(16.3)	82	(83.7)		90	(72.6)	82	(91.1)	8	(8.9)	
ypN category	139/0						0.134^b^	124/15						0.087^b^
pN0	44	(31.7)	7	(15.9)	37	(84.1)		38	(30.6)	30	(78.9)	8	(20.5)	
pN1	36	(25.9)	2	(5.6)	34	(94.4)		32	(25.8)	28	(87.5)	4	(12.5)	
pN2	35	(25.2)	5	(14.3)	30	(85.7)		32	(25.8)	28	(87.5)	4	(12.5)	
pN3 (a/b)	24	(17.3)	8	(33.3)	16	(66.7)		22	(17.7)	21	(95.5)	1	(4.5)	
ypN category	139/0						1.000^a^	124/15						0.156^a^
pN0	44	(31.7)	7	(15.9)	37	(84.1)		38	(30.6)	30	(78.9)	8	(21.1)	
pN +	95	(68.3)	15	(15.8)	80	(84.2)		86	(69.4)	77	(89.5)	9	(10.5)	
ypM category	139/0						0.015 ^b *^	124/15						0.374^b^
M0	128	(92.1)	17	(13.3)	111	(86.7)		116	(93.5)	99	(85.3)	17	(14.7)	
M1	11	(7.9)	5	(45.5)	6	(54.5)		8	(100)	8	(37.5)	0	(0.0)	
UICC stage	139/0						0.985^b^	124/15						0.009^b,^*
IA/IB	21	(15.1)	2	(9.5)	19	(90.5)		18	(14.5)	14	(77.8)	4	(22.2)	
IIA/IIB	25	(18.0)	6	(24.0)	19	(76.0)		22	(17.7)	16	(72.7)	6	(27.3)	
IIIA/IIIB/IIIC	76	(54.7)	11	(14.5)	65	(85.5)		69	(55.7)	62	(89.9)	7	(10.1)	
IV/IVA/IVB	17	(12.2)	3	(17.6)	14	(82.4)		15	(12.1)	15	(100)	0	(0.0)	
pR status	134/5						1.000^b^	120/19						0.125^b^
pR0	117	(87.3)	19	(16.2)	98	(83.8)		105	(87.5)	88	(83.8)	17	(16.2)	
pR1/pR2	17	(12.7)	3	(17.6)	14	(82.4)		15	(12.5)	15	(100)	0	(0.0)	
L category	136/3						0.627^b^	121/18						1.000^b^
L0	90	(66.2)	15	(16.7)	75	(83.3)		78	(64.5)	67	(85.9)	11	(14.1)	
L1	46	(33.8)	6	(13.0)	40	(87.0)		43	(35.5)	37	(86.0)	6	(14.0)	
V category	133/6						0.207^b^	118/21						0.357^b^
V0	122	(91.7)	20	(16.4)	102	(83.6)		107	(90.7)	91	(85.0)	16	(15.0)	
V1	11	(8.3)	8	(72.7)	3	(27.3)		11	(9.3)	11	(100)	0	(0.0)	
HER2 status	135/4						0.654^a^	122/17						0.604^a^
Negative	125	(92.6)	19	(15.2)	106	(84.8)		113	(92.6)	97	(85.8)	16	(14.2)	
Positive	10	(7.4)	2	(20.0)	8	(80.0)		9	(7.4)	9	(100)	0	(0.0)	
MET status	131/8						0.246^a^	117/22						0.527^a^
Negative	125	(95.4)	19	(15.2)	106	(84.8)		112	(95.7)	97	(86.6)	15	(13.4)	
Positive	6	(4.6)	2	(33.3)	4	(66.7)		5	(4.3)	4	(80.0)	1	(20.0)	
EBV status	120/19						1.000^a^	108/31						0.050^a,^*
Negative	117	(97.5)	20	(17.1)	97	(82.9)		105	(97.2)	92	(87.6)	13	(12.4)	
Positive	3	(2.5)	0	(0.0)	3	(100)		3	(2.8)	1	(33.3)	2	(66.7)	
MSI status	127/12						0.326^a^	115/24						0.055^a^
Negative (MSS)	120	(94.5)	19	(15.8)	101	(84.2)		108	(93.9)	95	(88.0)	13	(12.0)	
Positive (MSI)	7	(5.5)	2	(28.6)	5	(71.4)		7	(6.1)	4	(57.1)	3	(42.9)	
PD-L1 in tumor cells^c^	109/30						0.040^b,^*	100/39						0.001^b^
Negative (IRS = 0)	84	(77.1)	19	(22.6)	65	(77.4)		76	(76.0)	70	(92.1)	6	(7.9)	
Positive (IRS > 0)	25	(22.9)	1	(4.0)	24	(96.0)		24	(24.0)	15	(62.5)	9	(37.5)	
PD-L1 in immune cells^c^	113/26						0.001^b^	100/39						0.092^b^
Negative (QS ≤ 1)	66	(58.4)	19	(28.8)	47	(71.2)		55	(55.0)	50	(90.9)	5	(9.1)	
Positive (QS > 1)	47	(41.6)	2	(4.3)	45	(95.7)		45	(45.0)	35	(77.8)	10	(22.2)	
PD-1 in immune cells	113/26						0.612^a^	100/39						0.590^a^
Not present	7	(6.2)	2	(28.6)	5	(71.4)		7	(7.0)	7	(100.0)	0	(0.0)	
Present	106	(93.8)	19	(17.9)	87	(82.1)		93	(93.0)	78	(83.9)	15	(16.1)	

*IRS* immunoreactivity score, *QS* quantity score

*Statistically non-significant after multiple testing correction

^a^Fisher's exact test

^b^Kendall's tau test

^c^Cut-offs used by Schoop et al.

In NAT-GC (Table 3), LAG3 expression (dichotomized by adapted cut-off value) in TC was associated with Lauren phenotype (*p* < 0.001), presence of distant metastases (*p* = 0.015) and enrichment of PD-L1 + immune cells (*p* = 0.001). LAG3 + cell density in IM inversely correlated with ypT (*p* = 0.013) and UICC stage (*p* = 0.009). No associations were observed between LAG3 + cell infiltration and patient age, sex, tumor location, ypN, presence of LVI, and HER2 status. LAG3 expression in both compartments was associated with PD-L1 expression in tumor cells (*p* = 0.040, TC, and *p* = 0.001, IM). No significant associations were found between LAG3 expression and EBV, MSI, MET, and PD1 status, probably because of the limited number of cases. For analysis based on median values, see Suppl. Table 4.

## Discussion

Recently, LAG3 has gained increasing attention in immuno-oncology, and its putative role as a biomarker has been studied in various solid tumors. In this retrospective study of GC, we demonstrated the distribution patterns of LAG3 + cells within tumor tissues and their association with clinicopathological data and survival. The main findings of the current study are as follows: (1) LAG3 + immune cell density differs significantly between PR-GC and NAT-GC; (2) LAG3 + cell density shows significant differences in spatial distribution patterns between tumor compartments in PR-GC, which is not seen in NAT-GC; (3) CSS is significantly longer for patients with LAG3 expression above the calculated cut-offs in both cohorts; (4) LAG3 + immune cell density in TC is an independent prognostic factor of CSS in NAT-GC. Moreover, LAG3 + immune cell density is associated with various parameters, including sex, tumor location, Lauren phenotype, and HER2-, EBV-, MSI, and PD-1/PD-L1 status in PR-GC. In NAT-GC, an association with ypT and ypN categories, as well as PD-L1 expression in tumor cells and immune cells is observed.

High LAG3 + expression has been associated with poor prognosis in non-small cell lung cancer (He et al. 2017; Shepherd et al. 2022), renal cell cancer (Giraldo et al. 2015), hepatocellular cancer (Guo et al. 2020), and pancreatic cancer (Seifert et al. 2021) but with favorable prognosis in breast cancer (Burugu et al. 2017), colon cancer (Rhyner Agocs et al. 2021), and esophageal cancer (Zhang et al. 2018; Gebauer et al. 2020). In a large cohort of colorectal cancers, the prognostic effect differed based on the spatial location of LAG3 + tumor-infiltrating lymphocytes (TILs), and showed poor CSS in cases with high intra-tumoral LAG3 + TILs and improved CSS when LAG3 was identified in stromal immune cells (Al-Badran et al. 2021). Another interesting finding was an association of LAG3 with better outcome in early-stage tumors (Saleh et al. 2019). What is more, although LAG3 expression has been associated with more aggressive tumor features in breast cancer, patients with high LAG3 + intraepithelial TILs showed improved survival (Burugu et al. 2017). Interestingly, low LAG3 + expression became an independent predictor of favorable prognosis in breast cancers that were treated with neoadjuvant chemotherapy (Asano et al. 2022). To sum up, previous results suggest that the prognostic effect of LAG3 may depend on the tumor type, spatial location of TILs, clinical stage, and therapeutic approach (primary surgery *vs.* NAT).

LAG3 expression has also been observed in GC. A prospective study of solid tumors performed by Lee et al. (2019) included 53 metastatic GCs cases. They analyzed TMAs (two tissue cores from each GC) and regarded positive cases as any presence of LAG3 + immune cells regardless of their number (Lee et al. 2019). LAG3 expression was found in 24.7% of all GCs and was associated only with EBV status. No significant differences in sex, age, primary tumor site, Lauren classification, HER2 status, or OS were found between patients with or without LAG3 expression.

Lv et al. (2021) investigated the clinical and molecular correlations of LAG3 + cell infiltration in a large set of 464 GCs. Using TMAs (one core from each GC), they evaluated the mean density of LAG3 + immune cells and dichotomized all cases into LAG3 + low and LAG3 + high groups based on the median value of 59 cells/HPF. In their study, LAG3 + cell infiltration was associated with male sex and immuno-evasive contexture, and was found to be an independent adverse prognostic factor for both OS and disease-free survival (Lv et al. 2021). LAG3 showed higher expression in EBV + subtype and defective MLH1 subtype, as well as predicted poor survival in these subtypes (Lv et al. 2021).

Park et al. (2021) performed conventional and multiplex IHC on GC TMAs (*n* = 385, two tissue cores from each case) for immune cell markers and immune checkpoint receptors, including LAG3, as well as survival analysis. Positive LAG3 expression was defined as immunostaining in ≥ 5% of the immune cells, and it was found in 45.5%, 29.6%, and 50.1% of cases in the TC, IM, and TC or IM, respectively. LAG3 was expressed more commonly by CD3^+^/CD8^+^ T cells in the tumor area than in the stromal compartment (Park et al. 2021). It was associated with male sex, distal location, intestinal and mixed subtypes by Lauren, and better prognosis in multivariate survival analysis (Park et al. 2021).

These varying findings regarding the role of LAG3 in malignant tumors could be explained by the use of different assessment and scoring methods. Currently, there is no standardized assay for LAG3 expression in GC tissue. Several approaches, including absolute number per HPF (Lv et al. 2021) or per mm^2^ (Shepherd et al. 2022), and cut-offs, such as any positivity (Burugu et al. 2017; Lee et al. 2019; Rhyner Agocs et al. 2021), median number (Lv et al. 2021), 1% of all nucleated cells (Johnson et al. 2022), or 5% of all TILs (Park et al. 2021), have been used in GC and other solid malignancies. Furthermore, the use of the TMA technique might influence the results because of the limited amount of tissue, uneven distribution of infiltrating immune cells, and heterogeneous expression of immune checkpoint molecules. It has been demonstrated on TMAs as substitutes for core biopsies that at least five biopsies are needed to reflect the objective status of PD-L1 expression as in whole sections of GC (Ye et al. 2020).

In previous studies, LAG3 expression was correlated with EBV + and MSI-H GC molecular subtypes. Our results confirm this association in a Western GC cohort, however only when using the median cut-off values (an approach used by Lv et al.). Both subtypes are known to contain a high number of TILs. DNA mismatch repair-deficient tumors harbor large amounts of somatic mutations and tumor-specific neo-antigens that trigger neo-antigen-specific T cells and make them sensitive to immune checkpoint blockade (Le et al. 2017). Similarly, EBV evokes an active immune response that leads to enrichment in activated CD8 + T cells (Landais et al. 2005; van Beek et al. 2006). In a study of Hodgkin’s lymphoma, EBV infection increased gene expression of *LAG3* and immunosuppressive cytokines associated with type-1 T regulatory cells (Tr1) (Morales et al. 2014).

Although expression of inhibitory receptors is a hallmark of T cell exhaustion, they are transiently expressed already on activated effector T cells (Wherry and Kurachi 2015). According to a study by Bae et al. (2014), the majority of LAG3 is localized in lysosomes in resting cells and translocates to the cell surface upon stimulation. This could explain the enrichment of LAG3 + cells in early-invasive tumors (pT1/2) despite LAG3 classical role in immunosuppressive TME. We assume that, in such cases, LAG3 expression could rather be a sign of an activated immune response. This feature makes the LAG3 protein an attractive marker for the assessment of potential anti-tumor response and, thus, patient stratification for immunotherapy.

Another interesting finding is higher LAG3 expression in males, which was also demonstrated by both larger Asian studies (Park et al. 2021; Lv et al. 2021). There is increasing awareness about sexual dimorphism in the immune response in solid cancers and its effect on patient outcomes. In a meta-analysis of 17 clinical trials, males appeared to benefit more frequently from ICI therapy than females (Parmar et al. 2022). Higher tumor mutational burden/antigenicity and T cell-dominating inflammation are thought to be key features of such differences in males (Conforti et al. 2019). Previously, tumor-associated neutrophils at the invasive front were found to be an independent predictor of CSS in females of the same PR-GC cohort (Clausen et al. 2020). Current findings expand the sex-based differences in TME of PR-GC, now also including LAG3.

Furthermore, NAT also affects TME. Chemotherapeutics and anticancer agents increase tumor antigenicity and response of CTLs or inhibit immunosuppressive pathways, thus favoring anti-tumor response (Galluzzi et al. 2015). Previous GC studies of paired pre-NAT biopsies and post-NAT resection specimens demonstrated that chemotherapy increases CD8 + T cell density (Yu et al. 2019; Wei et al. 2021; Christina Svensson et al. 2021). It also induces macrophage markers CD68 and CD163 (Wei et al. 2021) and immune checkpoint molecules PD1, PD-L1, and TIM3 (Yu et al. 2019), but decreases CD20 + B cell density (Christina Svensson et al. 2021). However, there are no data regarding LAG3 in GC. Previous studies on esophageal and rectal cancers revealed upregulation of immune checkpoint molecules, including LAG3, after chemo-radiation (Kelly et al. 2018; Peng et al. 2021). Unfortunately, we were unable to characterize the direct effect of NAT on the TME. However, in our study, the median density of LAG3 + cells was lower in NAT-GC than that in PR-GC. There was no significant difference in LAG3 + cell distribution between TC and IM and among molecular subtypes (i.e., EBV and MSI) in NAT-GC, as observed in PR-GC. Collectively, these findings support the notion that NAT also affects the expression of LAG3 in GC.

Our study has several limitations. This retrospective study was restricted to immunohistochemical assessment of LAG3. No further subtyping of LAG3 + immune cells was performed. In the NAT-GC sub-cohort, different treatment schemes and doses were used (according to the changing treatment protocols). No matched pre- and post-treatment samples were compared. Digital analysis is performed only on sections where marking of tumor compartments was possible. Some samples lacked IM values because the stained sections included neoplastic tissue only (marked as TC). However, this is a large and well-characterized set of both PR-GC and NAT-GC. To our knowledge, this is the first study to investigate LAG3 expression in GC of Caucasian origin. The assessment was performed on whole tissue sections to reduce the under- or over-representation of LAG3 + cells due to their heterogeneous distribution within the TME. The same marking techniques and software settings were used for the entire cohort. To increase the reproducibility of the results, a dichotomous scoring method was used.

In conclusion, despite the postulated immunosuppressive role of LAG3 within the TME of solid tumors, the current findings demonstrate differences in the TME among the main histological and molecular subtypes of GC. A high density of LAG3 + cells predicts favorable prognosis in NAT-GC. LAG3 may have different, stage-based functional roles within the TME. Increased numbers of LAG3 + cells within GC tissue could be a sign of crosstalk between cancer and immune cells rather than a sign of exhausted, dysfunctional T cells. The precise mechanisms by which LAG3 regulates T cell function require further investigation. The current findings also raise an important issue in GC immune checkpoint studies based on TMA analysis using single-tissue cores. In the present study, LAG3 + immune cells were detectable in all cases and demonstrated spatial heterogeneity. Considering the popularity of LAG3 as a potential biomarker in cancer studies, a robust LAG3 assay should be developed. Cut-offs may vary between TC and IM, as well as between primarily resected and neo-adjuvantly treated GCs, which merits specific consideration. Matters are far more complicated than anticipated.

## Supplementary Information

Below is the link to the electronic supplementary material.

Supplementary file1 (DOCX 560 kb)

## Data Availability

The datasets generated during and/or analyzed during the current study are available from the corresponding author on reasonable request.

## Abbreviations

CI: Confidence interval
CSS: Cancer-specific survival
CTLA4: Cytotoxic T-lymphocyte-associated protein 4
CTLs: Cytotoxic T lymphocytes
EBV: Epstein–Barr virus
GC: Adenocarcinoma of the stomach and gastroesophageal junction; gastric cancer
ICI: Immune checkpoint inhibitors
IM: Invasive margin
LAG3: Lymphocyte activation gene 3
LVI: Lymphovascular invasion
MSI: Microsatellite instability
NAT-GC: Neoadjuvantly treated gastric cancer
OS: Overall survival
PD-1: Programmed cell death 1
PD-L1: Programmed cell death 1 ligand
pR: Status of resection lines
PR-GC: Primarily resected gastric cancer
TC: Tumor center
TILs: Tumor-infiltrating lymphocytes
TMAs: Tissue microarrays
TME: Tumor microenvironment

## References

[CR1] Al-Badran SS, Grant L, Campo MV (2021). Relationship between immune checkpoint proteins, tumour microenvironment characteristics, and prognosis in primary operable colorectal cancer. J Pathol Clin Res.

[CR2] Anderson AC, Joller N, Kuchroo VK (2016). Lag-3, Tim-3, and TIGIT: co-inhibitory receptors with specialized functions in immune regulation. Immunity.

[CR3] Andrews LP, Marciscano AE, Drake CG, Vignali DAA (2017). LAG3 (CD223) as a cancer immunotherapy target. Immunol Rev.

[CR4] Arnold M, Rutherford M, Lam F, et al (2019) ICBP SURVMARK-2 online tool: International Cancer Survival Benchmarking. http://gco.iarc.fr/survival/survmark. Accessed 1 Mar 2023

[CR5] Asano Y, Kashiwagi S, Takada K (2022). Clinical significance of expression of immunoadjuvant molecules (LAG-3, TIM-3, OX-40) in neoadjuvant chemotherapy for breast cancer. Anticancer Res.

[CR6] Bae J, Lee SJ, Park C-G (2014). Trafficking of LAG-3 to the surface on activated T cells via its cytoplasmic domain and protein kinase C signaling. J Immunol.

[CR7] Baixeras E, Huard B, Miossec C (1992). Characterization of the lymphocyte activation gene 3-encoded protein. A new ligand for human leukocyte antigen class II antigens. J Exp Med.

[CR8] Benjamini Y, Hochberg Y (1995). Controlling the false discovery rate: a practical and powerful approach to multiple testing. J R Stat Soc Ser B (methodol).

[CR9] Blank CU, Haining WN, Held W (2019). Defining “T cell exhaustion”. Nat Rev Immunol.

[CR10] Böger C, Behrens H-M, Mathiak M (2016). PD-L1 is an independent prognostic predictor in gastric cancer of Western patients. Oncotarget.

[CR11] Böger C, Krüger S, Behrens HM (2017). Epstein-Barr virus-associated gastric cancer reveals intratumoral heterogeneity of PIK3CA mutations. Ann Oncol.

[CR12] Brierley JD, Gospodarowicz MK, Wittekind C (2017). TNM classification of malignant tumours.

[CR13] Budczies J, Klauschen F, Sinn BV (2012). Cutoff Finder: a comprehensive and straightforward Web application enabling rapid biomarker cutoff optimization. PLoS One.

[CR14] Burugu S, Gao D, Leung S (2017). LAG-3+ tumor infiltrating lymphocytes in breast cancer: clinical correlates and association with PD-1/PD-L1+ tumors. Ann Oncol.

[CR15] Christina Svensson M, Lindén A, Nygaard J (2021). T cells, B cells, and PD-L1 expression in esophageal and gastric adenocarcinoma before and after neoadjuvant chemotherapy: relationship with histopathological response and survival. Oncoimmunology.

[CR16] Clausen F, Behrens H-M, Krüger S, Röcken C (2020). Sexual dimorphism in gastric cancer: tumor-associated neutrophils predict patient outcome only for women. J Cancer Res Clin Oncol.

[CR17] Conforti F, Pala L, Bagnardi V (2019). Sex-based differences of the tumor mutational burden and T-cell inflammation of the tumor microenvironment. Ann Oncol.

[CR18] Galluzzi L, Vacchelli E, Bravo-San Pedro J-M (2014). Classification of current anticancer immunotherapies. Oncotarget.

[CR19] Galluzzi L, Buqué A, Kepp O (2015). Immunological effects of conventional chemotherapy and targeted anticancer agents. Cancer Cell.

[CR20] Gebauer F, Krämer M, Bruns C (2020). Lymphocyte activation gene-3 (LAG3) mRNA and protein expression on tumour infiltrating lymphocytes (TILs) in oesophageal adenocarcinoma. J Cancer Res Clin Oncol.

[CR21] Giraldo NA, Becht E, Pagès F (2015). Orchestration and prognostic significance of immune checkpoints in the microenvironment of primary and metastatic renal cell cancer. Clin Cancer Res.

[CR22] Guo M, Yuan F, Qi F (2020). Expression and clinical significance of LAG-3, FGL1, PD-L1 and CD8(+)T cells in hepatocellular carcinoma using multiplex quantitative analysis. J Transl Med.

[CR23] Guy C, Mitrea DM, Chou P-C (2022). LAG3 associates with TCR-CD3 complexes and suppresses signaling by driving co-receptor-Lck dissociation. Nat Immunol.

[CR24] He X, Xu C (2020). Immune checkpoint signaling and cancer immunotherapy. Cell Res.

[CR25] He Y, Yu H, Rozeboom L (2017). LAG-3 protein expression in non-small cell lung cancer and its relationship with PD-1/PD-L1 and tumor-infiltrating lymphocytes. J Thorac Oncol.

[CR26] Hendry S, Salgado R, Gevaert T (2017). Assessing tumor-infiltrating lymphocytes in solid tumors: a practical review for pathologists and proposal for a standardized method from the international immunooncology biomarkers working group: part 1: assessing the host immune response, TILs in invasive breast carcinoma and ductal carcinoma in situ, metastatic tumor deposits and areas for further research. Adv Anat Pathol.

[CR27] Huang C-T, Workman CJ, Flies D (2004). Role of LAG-3 in regulatory T cells. Immunity.

[CR28] Johnson L, McCune B, Locke D (2022). Development of a LAG-3 immunohistochemistry assay for melanoma. J Clin Pathol.

[CR29] Kelly RJ, Zaidi AH, Smith MA (2018). The dynamic and transient immune microenvironment in locally advanced esophageal adenocarcinoma post chemoradiation. Ann Surg.

[CR30] Kouo T, Huang L, Pucsek AB (2015). Galectin-3 shapes antitumor immune responses by suppressing CD8+ T cells via LAG-3 and inhibiting expansion of plasmacytoid dendritic cells. Cancer Immunol Res.

[CR31] Landais E, Saulquin X, Houssaint E (2005). The human T cell immune response to Epstein–Barr virus. Int J Dev Biol.

[CR32] Le DT, Durham JN, Smith KN (2017). Mismatch repair deficiency predicts response of solid tumors to PD-1 blockade. Science.

[CR33] Lee SJ, Byeon S-J, Lee J (2019). LAG3 in solid tumors as a potential novel immunotherapy target. J Immunother.

[CR34] Lv K, Li R, Cao Y (2021). Lymphocyte-activation gene 3 expression associates with poor prognosis and immunoevasive contexture in Epstein–Barr virus-positive and MLH1-defective gastric cancer patients. Int J Cancer.

[CR35] Maeda TK, Sugiura D, Okazaki I-M (2019). Atypical motifs in the cytoplasmic region of the inhibitory immune co-receptor LAG-3 inhibit T cell activation. J Biol Chem.

[CR36] Mathiak M, Warneke VS, Behrens H-M (2017). Clinicopathologic characteristics of microsatellite instable gastric carcinomas revisited: urgent need for standardization. Appl Immunohistochem Mol Morphol.

[CR37] Metzger M-L, Behrens H-M, Böger C (2016). MET in gastric cancer—discarding a 10% cutoff rule. Histopathology.

[CR38] Morales O, Mrizak D, François V (2014). Epstein–Barr virus infection induces an increase of T regulatory type 1 cells in Hodgkin lymphoma patients. Br J Haematol.

[CR39] National Cancer Institute SEER*Explorer: an interactive website for SEER cancer statistics. https://seer.cancer.gov/explorer/. Accessed 1 Mar 2023

[CR40] Paik J (2022). Nivolumab plus relatlimab: first approval. Drugs.

[CR41] Park Y, Seo AN, Koh J (2021). Expression of the immune checkpoint receptors PD-1, LAG3, and TIM3 in the immune context of stage II and III gastric cancer by using single and chromogenic multiplex immunohistochemistry. Oncoimmunology.

[CR42] Parmar K, Subramanyam S, Attwood K (2022). Anti PD-1/anti PDL-1 inhibitors in advanced gastroesophageal cancers: a systematic review and meta-analysis of phase 2/3 randomized controlled trials. Pharmaceutics.

[CR43] Peng Q-Q, Li J-L, Xin P-L (2021). Assessment of the expression and response of PD-1, LAG-3, and TIM-3 after neoadjuvant radiotherapy in rectal cancer. Neoplasma.

[CR44] Rhyner Agocs G, Assarzadegan N, Kirsch R (2021). LAG-3 expression predicts outcome in stage II colon cancer. J Pers Med.

[CR45] Saleh RR, Peinado P, Fuentes-Antrás J (2019). Prognostic value of lymphocyte-activation gene 3 (LAG3) in cancer: a meta-analysis. Front Oncol.

[CR46] Schoop H, Bregenzer A, Halske C (2020). Therapy resistance in neoadjuvantly treated gastric cancer and cancer of the gastroesophageal junction is associated with an increased expression of immune checkpoint inhibitors-comparison against a therapy naïve cohort. Transl Oncol.

[CR47] Seifert L, Plesca I, Müller L (2021). LAG-3-expressing tumor-infiltrating T cells are associated with reduced disease-free survival in pancreatic cancer. Cancers (basel).

[CR48] Shan C, Li X, Zhang J (2020). Progress of immune checkpoint LAG-3 in immunotherapy. Oncol Lett.

[CR49] Shepherd DJ, Tabb ES, Kunitoki K (2022). Lymphocyte-activation gene 3 in non-small-cell lung carcinomas: correlations with clinicopathologic features and prognostic significance. Mod Pathol.

[CR50] Shergold AL, Millar R, Nibbs RJB (2019). Understanding and overcoming the resistance of cancer to PD-1/PD-L1 blockade. Pharmacol Res.

[CR51] Sung H, Ferlay J, Siegel RL (2021). Global cancer statistics 2020: GLOBOCAN estimates of incidence and mortality worldwide for 36 cancers in 185 countries. CA Cancer J Clin.

[CR52] Tawbi HA, Schadendorf D, Lipson EJ (2022). Relatlimab and nivolumab versus nivolumab in untreated advanced melanoma. N Engl J Med.

[CR53] Triebel F, Jitsukawa S, Baixeras E (1990). LAG-3, a novel lymphocyte activation gene closely related to CD4. J Exp Med.

[CR54] van Beek J, zur Hausen A, Snel SN (2006). Morphological evidence of an activated cytotoxic T-cell infiltrate in EBV-positive gastric carcinoma preventing lymph node metastases. Am J Surg Pathol.

[CR55] van den Bulk J, Verdegaal EM, de Miranda NF (2018). Cancer immunotherapy: broadening the scope of targetable tumours. Open Biol.

[CR56] Wang J, Sanmamed MF, Datar I (2019). Fibrinogen-like protein 1 is a major immune inhibitory ligand of LAG-3. Cell.

[CR57] Warneke VS, Behrens H-M, Böger C (2013). Her2/neu testing in gastric cancer: evaluating the risk of sampling errors. Ann Oncol.

[CR58] Wei Q, Xu Q, Yuan X (2021). Immunological impact of chemotherapy on the tumor microenvironment in gastric cancer. J Surg Oncol.

[CR59] Wherry EJ, Kurachi M (2015). Molecular and cellular insights into T cell exhaustion. Nat Rev Immunol.

[CR60] Woo S-R, Turnis ME, Goldberg MV (2012). Immune inhibitory molecules LAG-3 and PD-1 synergistically regulate T-cell function to promote tumoral immune escape. Cancer Res.

[CR61] Workman CJ, Dugger KJ, Vignali DAA (2002). Cutting edge: molecular analysis of the negative regulatory function of lymphocyte activation gene-3. J Immunol.

[CR62] Xu F, Liu J, Liu D (2014). LSECtin expressed on melanoma cells promotes tumor progression by inhibiting antitumor T-cell responses. Cancer Res.

[CR63] Ye M, Huang D, Zhang Q (2020). Heterogeneous programmed death-ligand 1 expression in gastric cancer: comparison of tissue microarrays and whole sections. Cancer Cell Int.

[CR64] Yu Y, Ma X, Zhang Y (2019). Changes in expression of multiple checkpoint molecules and infiltration of tumor immune cells after neoadjuvant chemotherapy in gastric cancer. J Cancer.

[CR65] Zhang Y, Zheng J (2020). Functions of immune checkpoint molecules beyond immune evasion. Adv Exp Med Biol.

[CR66] Zhang Y, Liu Y-D, Luo Y-L (2018). Prognostic value of lymphocyte activation gene-3 (LAG-3) expression in esophageal squamous cell carcinoma. J Cancer.

