# Supplementary material for: LAG3 in gastric cancer: it’s complicated
Source: J Cancer Res Clin Oncol. 2023 Jun 14;149(12):10797–811. doi: 10.1007/s00432-023-04954-1 (PMC10423140; doi:10.1007/s00432-023-04954-1)
Supplement: Supplementary file 1 — Supplementary file1 (DOCX 560 kb) [file 432_2023_4954_MOESM1_ESM.docx]

**Supplement table 1** Characteristics of both cohorts

| **Characteristics** | **Primary resected**  **gastric cancer**  **(PR-GC)** | | **Neoadjuvant-treated gastric cancer**  **(NAT-GC)** | |
| --- | --- | --- | --- | --- |
|  | **n,**  **valid/missing** | **(%)** | **n,**  **valid/missing** | **(%)** |
| Total cases | 441 |  | 139 |  |
| Median age (range) | 68 (28 – 92) |  | 64 (21 – 82) |  |
| Sex | 441/0 |  | 139/0 |  |
| Male | 277 | (62.8) | 29 | (20.9) |
| Female | 164 | (37.2) | 110 | (79.1) |
| Location | 438/3 |  | 139/0 |  |
| Proximal stomach | 141 | (32.2) | 99 | (71.2) |
| Distal stomach | 297 | (67.8) | 40 | (28.8) |
| Laurén phenotype | 441/0 |  | 139/0 |  |
| Intestinal | 226 | (51.2) | 73 | (52.5) |
| Diffuse | 138 | (31.3) | 27 | (19.4) |
| Mixed | 29 | (6.6) | 29 | (20.9) |
| Unclassified | 48 | (10.9) | 48 | (7.2) |
| (y) pT category | 441/0 |  | 139/0 |  |
| T1a / T1b | 55 | (12.5) | 18 | (13.0) |
| T2 | 48 | (10.9) | 23 | (16.5) |
| T3 | 181 | (41.0) | 88 | (63.3) |
| T4a / T4b | 157 | (35.6) | 10 | (7.2) |
| (y) pN category | 440/1 |  | 139/0 |  |
| N0 | 127 | (28.9) | 45 | (32.4) |
| N1 | 60 | (13.6) | 36 | (25.9) |
| N2 | 77 | (17.5) | 34 | (24.4) |
| N3a / N3b | 176 | (40.0) | 24 | (17.3) |
| pM category | 441/0 |  | 139/0 |  |
| M0 | 358 | (81.2) | 128 | (92.1) |
| M1 | 83 | (18.8) | 11 | (7.9) |
| UICC stage | 440/1 |  | 139/0 |  |
| IA / IB | 75 | (17.0) | 21 | (15.1) |
| IIA / IIB | 96 | (21.8) | 25 | (18.0) |
| IIIA / IIIB / IIIC | 186 | (42.3) | 76 | (54.7) |
| IV | 83 | (18.9) | 17 | (12.2) |
| pR status | 436/5 |  | 134/5 |  |
| pR0 | 381 | (87.4) | 117 | (87.3) |
| pR1 / pR2 | 55 | (12.6) | 17 | (12.7) |
| L category | 421/20 |  | 136/3 |  |
| L0 | 203 | (48.2) | 90 | (66.2) |
| L1 | 218 | (51.8) | 46 | (33.8) |
| V category | 420/21 |  | 133/6 |  |
| V0 | 373 | (88.8) | 122 | (91.7) |
| V1 | 47 | (11.2) | 11 | (8.3) |
| Helicobacter pylori status | 374/67 |  | NA |  |
| Negative | 317 | (84.8) | NA |  |
| Positive | 57 | (15.2) | NA |  |
| EBV status | 431/10 |  | 120/19 |  |
| Negative | 412 | (95.6) | 117 | (97.5) |
| Positive | 19 | (4.4) | 3 | (2.5) |
| HER2 status | 412/29 |  | 135/4 |  |
| Negative | 378 | (91.7) | 125 | (92.6) |
| Positive | 34 | (8.3) | 10 | (7.4) |
| cMET status | 430/11 |  | 131/8 |  |
| Negative | 397 | (92.3) | 125 | (95.4) |
| Positive | 33 | (7.7) | 6 | (4.6) |
| MSI status | 429/12 |  | 127/12 |  |
| Negative (MSS) | 397 | (92.5) | 120 | (94.5) |
| Positive (MSI) | 32 | (7.5) | 7 | (5.5) |
| PD-L1 in tumour cells * | 419/22 |  | 109/30 |  |
| Negative | 319 | (76.1) | 84 | (77.1) |
| Positive | 100 | (23.9) | 25 | (22.9) |
| PD-L1 in immune cells | 419/22 |  | 113/26 |  |
| Negative (QS ≤ 1) | 267 | (63.7) | 66 | (58.4) |
| Positive (QS > 1) | 152 | (36.3) | 47 | (41.6) |
| PD-1 in immune cells | 422/19 |  | 113/26 |  |
| Not present | 191 | (45.3) | 7 | (6.2) |
| Present | 231 | (54.7) | 106 | (93.8) |

UICC, Union for International Cancer Control; NA, not available; * divided by median immunoreactivity score (used by Böger et al. and Schoop et al.); QS, quantity score (used by Böger et al. and Schoop et al.).

**Supplement table 2** Survival data among LAG3 expression groups based on median and adjusted cut-offs of LAG3+ cell densities

| **Subgroup** | **Total / events / censored** | **Survival, months** | | ***p*-value**  **(log rank)** |
| --- | --- | --- | --- | --- |
|  |  | **Median** | **(95% CI)** |  |
| **Primary resected GC** |  |  |  |  |
| ***Cancer-specific survival*** ^a^ |  |  |  |  |
| Tumour center |  |  |  |  |
| LAG3 low (< 55.15 cells/mm^2^) | 202 / 144 / 58 | 14.2 | (11.5 – 16.8) | 0.149 |
| LAG3 high (≥ 55.15) | 197 / 130 / 67 | 18.1 | (12.2 – 24.0) |  |
| Invasive margin |  |  |  |  |
| LAG3 low (< 70.35) | 184 / 124 / 60 | 15.5 | (12.1 – 18.8) | 0.602 |
| LAG3 high (≥ 70.35) | 184 / 128 / 56 | 17.9 | (11.7 – 24.0) |  |
| ***Cancer-specific survival*** ^b^ |  |  |  |  |
| Tumour center |  |  |  |  |
| LAG3 low (< 21.45) | 69 / 52 / 17 | 10.1 | (7.5 – 12.7) | 0.008^*^ |
| LAG3 high (≥ 21.45) | 330 / 222 / 108 | 17.9 | (13.7 – 22.1) |  |
| Invasive margin |  |  |  |  |
| LAG3 low (< 208.50) | 311 / 220 / 91 | 14.7 | (12.1 – 17.2) | 0.006 |
| LAG3 high (≥ 208.50) | 57 / 32 / 25 | 33.8 | (21.4 – 46.3) |  |
| **Neoadjuvant treated GC** |  |  |  |  |
| ***Cancer-specific survival*** ^a^ |  |  |  |  |
| Tumour center |  |  |  |  |
| LAG3 low (< 41.37 cells/mm^2^) | 64 / 36 / 28 | 19.9 | (10.6 – 29.2) | 0.340 |
| LAG3 high (≥ 41.37) | 66 / 33 / 33 | 27.2 | (19.2 – 35.2) |  |
| Invasive margin |  |  |  |  |
| LAG3 low (< 43.28) | 58 / 34 / 24 | 13.7 | (6.8 – 20.5) | 0.111 |
| LAG3 high (≥ 43.28) | 58 / 30 / 28 | 31.8 | (25.1 – 38.5) |  |
| ***Cancer-specific survival*** ^b^ |  |  |  |  |
| Tumour center |  |  |  |  |
| LAG3 low (< 12.62) | 22 / 16 / 6 | 13.2 | (5.6 – 20.8) | 0.003 |
| LAG3 high (≥ 12.62) | 108 / 53 / 55 | 27.3 | (19.6 – 35.1) |  |
| Invasive margin |  |  |  |  |
| LAG3 low (< 123.00) | 100 / 59 / 41 | 22.4 | (15.5 – 29.2) | 0.136 |
| LAG3 high (≥ 123.00) | 16 / 5 / 11 | 28.0 | (NA) |  |

^a^ based on the median value; ^b^ based on the adjusted cut-off value of *Cutoff Finder*; CI, confidence interval; GC, gastric cancer; NA, not applicable; ^*^ statistically non-significant after multiple testing correction.

**
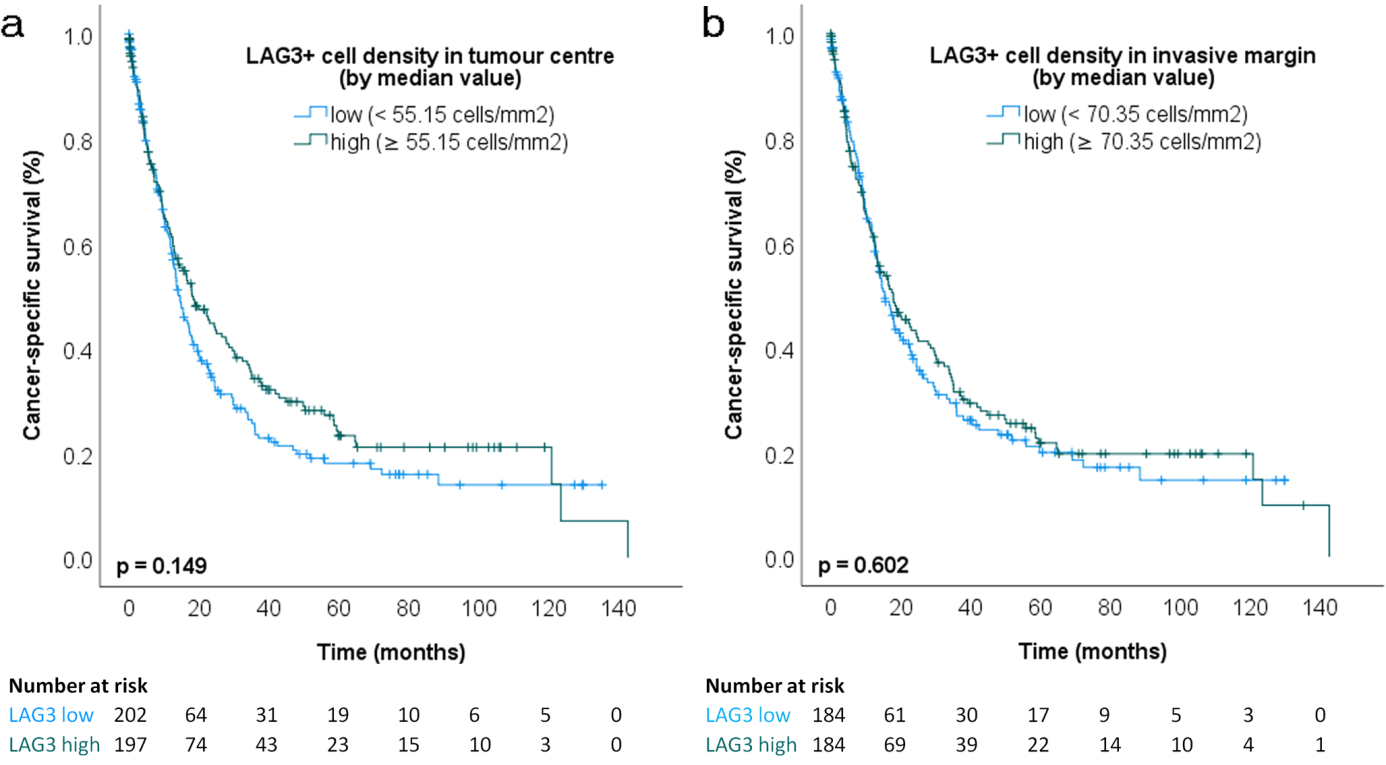
**

**Supplement figure 1** Kaplan-Meier curves in primary resected gastric cancer: cancer-specific survival according to LAG3+ cell density groups splitted by median (**a** tumour centre, p = 0.149, log-rank test; **b** invasive margin, p = 0.602, log-rank test; small vertical lines in the graph indicate censored data)


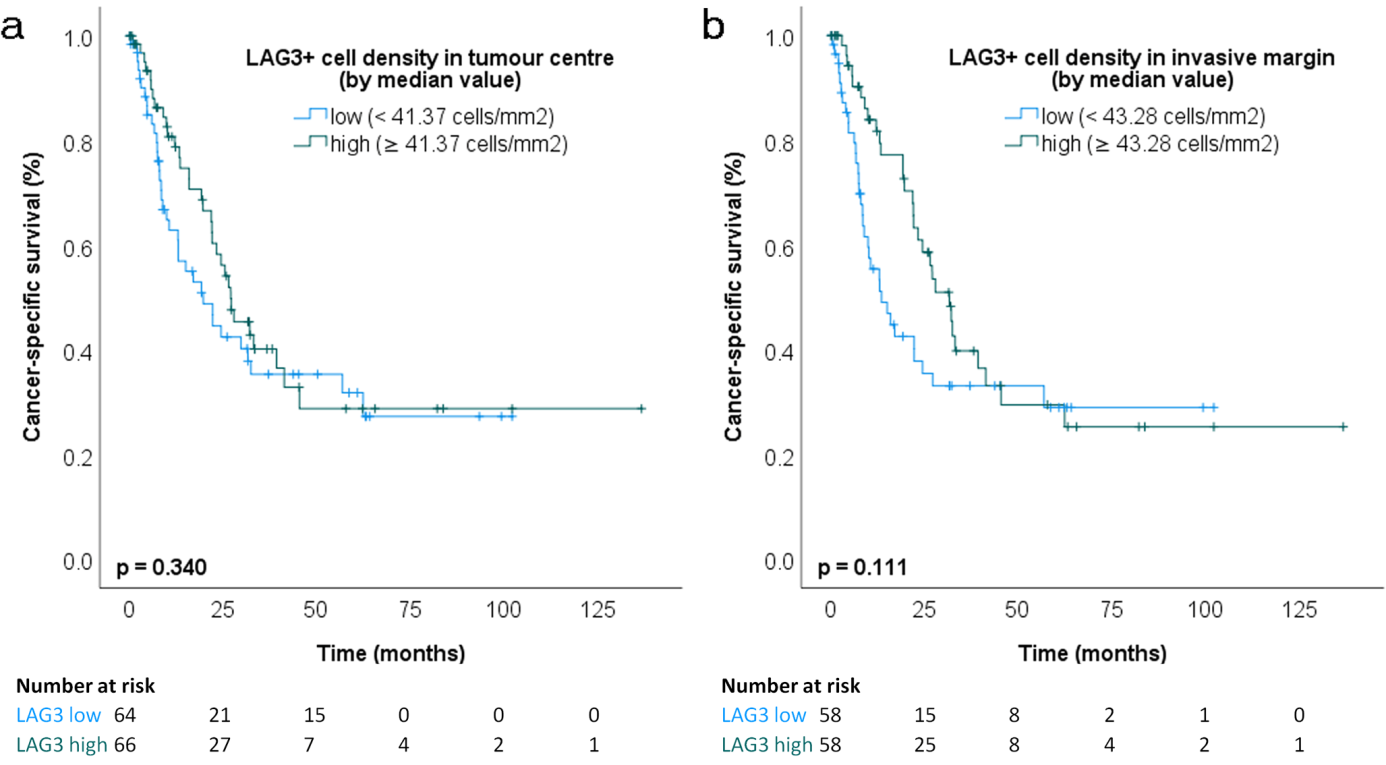


**Supplement figure 2** Kaplan-Meier curves in neoadjuvant treated gastric cancer: cancer-specific survival according to LAG3+ cell density groups splitted by median (a tumour centre, p = 0.340, log-rank test; b invasive margin, p = 0.111, log-rank test; small vertical lines in the graph indicate censored data)

**Supplement table 3** Primary-resected GC: Association of LAG3+ cell density (dichotomized by median) with demographical and clinicopathological patient characteristics

|  | **Tumour center** | | | | | | | **Invasive margin** | | | | | | |
| --- | --- | --- | --- | --- | --- | --- | --- | --- | --- | --- | --- | --- | --- | --- |
| **Characteristics** | **Valid/missing** | | **LAG3 low** | | **LAG3 high** | | ***p*-value** | **Valid/missing** | | **LAG3 low** | | **LAG3 high** | | ***p*-value** |
|  | **n** | **(%)** | **n** | **(%)** | **n** | **(%)** |  | **n** | **(%)** | **n** | **(%)** | **n** | **(%)** |  |
| Sex | 441/0 |  |  |  |  |  | 0.140 ^a^ | 408/33 |  |  |  |  |  | 0.002 ^a^ |
| Male | 277 | (62.8) | 131 | (47.3) | 146 | (52.7) |  | 254 | (62.3) | 111 | (43.7) | 143 | (56.3) |  |
| Female | 164 | (37.2) | 90 | (54.9) | 74 | (45.1) |  | 154 | (37.7) | 93 | (60.4) | 61 | (39.6) |  |
| Age | 441/0 |  |  |  |  |  | 0.704 ^b^ | 408/33 |  |  |  |  |  | 0.552 ^b^ |
| < 68 years | 220 | (49.9) | 108 | (49.1) | 112 | (50.9) |  | 201 | (49.3) | 104 | (51.7) | 97 | (48.3) |  |
| ≥ 68 years | 221 | (50.1) | 113 | (51.1) | 108 | (48.9) |  | 207 | (50.7) | 100 | (48.3) | 107 | (51.7) |  |
| Location | 438/3 |  |  |  |  |  | 0.358 ^a^ | 405/36 |  |  |  |  |  | 0.025 ^a *^ |
| Proximal stomach | 141 | (32.2) | 66 | (46.8) | 75 | (53.2) |  | 130 | (32.1) | 54 | (41.5) | 76 | (58.5) |  |
| Distal stomach | 297 | (67.8) | 154 | (51.9) | 143 | (48.1) |  | 275 | (67.9) | 148 | (53.8) | 127 | (46.2) |  |
| Laurén phenotype | 441/0 |  |  |  |  |  | 0.001 ^a^ | 408/33 |  |  |  |  |  | < 0.001 ^a^ |
| Intestinal | 226 | (51.2) | 113 | (50.0) | 113 | (50.0) |  | 216 | (52.9) | 97 | (44.9) | 119 | (55.1) |  |
| Diffuse | 138 | (31.3) | 83 | (60.1) | 55 | (39.9) |  | 119 | (29.2) | 82 | (68.9) | 37 | (31.1) |  |
| Mixed | 29 | (6.6) | 12 | (41.4) | 17 | (58.6) |  | 26 | (6.4) | 12 | (46.2) | 14 | (53.8) |  |
| Unclassified | 48 | (10.9) | 13 | (27.1) | 35 | (72.9) |  | 47 | (11.5) | 13 | (27.7) | 34 | (72.3) |  |
| pT category | 441/0 |  |  |  |  |  | 0.075 ^b^ | 408/33 |  |  |  |  |  | 0.640 ^b^ |
| pT1 (a/b) | 55 | (12.5) | 22 | (40.0) | 33 | (60.0) |  | 48 | (11.8) | 25 | (52.1) | 23 | (47.9) |  |
| pT2 | 48 | (10.9) | 16 | (33.3) | 32 | (66.7) |  | 47 | (11.5) | 18 | (38.3) | 29 | (61.7) |  |
| pT3 | 181 | (41.0) | 102 | (56.4) | 79 | (43.6) |  | 172 | (42.2) | 90 | (52.3) | 82 | (47.7) |  |
| pT4 (a/b) | 157 | (35.6) | 81 | (51.6) | 76 | (48.4) |  | 141 | (34.5) | 71 | (50.4) | 70 | (49.6) |  |
| pT category | 441/0 |  |  |  |  |  | 0.002 ^a^ | 408/33 |  |  |  |  |  | 0.349 ^a^ |
| pT1 (a/b) / pT2 | 103 | (23.4) | 38 | (36.9) | 65 | (63.1) |  | 95 | (23.3) | 43 | (45.3) | 52 | (54.7) |  |
| pT3 / pT4 (a/b) | 338 | (76.6) | 183 | (54.1) | 155 | (45.9) |  | 313 | (76.7) | 161 | (51.4) | 152 | (48.6) |  |
| pN category | 440/1 |  |  |  |  |  | 0.079 ^b^ | 408/33 |  |  |  |  |  | 0.692 ^b^ |
| pN0 | 127 | (28.9) | 54 | (42.5) | 73 | (57.5) |  | 120 | (29.4) | 62 | (51.7) | 58 | (48.3) |  |
| pN1 | 60 | (13.6) | 33 | (55.0) | 27 | (45.0) |  | 57 | (14.0) | 29 | (50.9) | 28 | (49.1) |  |
| pN2 | 77 | (17.5) | 39 | (50.6) | 38 | (49.4) |  | 69 | (16.9) | 33 | (47.8) | 36 | (52.2) |  |
| pN3 (a/b) | 176 | (40.0) | 95 | (54.0) | 81 | (46.0) |  | 162 | (39.7) | 80 | (49.4) | 82 | (50.6) |  |
| pN category | 440/1 |  |  |  |  |  | 0.046 ^a *^ | 408/33 |  |  |  |  |  | 0.745 ^a^ |
| pN0 | 127 | (28.9) | 54 | (42.5) | 73 | (57.5) |  | 120 | (29.4) | 62 | (51.7) | 58 | (48.3) |  |
| pN+ | 313 | (71.1) | 167 | (53.4) | 146 | (46.6) |  | 288 | (70.6) | 142 | (49.3) | 146 | (50.7) |  |
| pM category | 441/0 |  |  |  |  |  | 0.808 ^b^ | 408/33 |  |  |  |  |  | 0.606 ^b^ |
| M0 | 358 | (81.2) | 178 | (49.7) | 180 | (50.3) |  | 335 | (82.1) | 165 | (49.3) | 170 | (50.7) |  |
| M1 | 83 | (18.8) | 43 | (51.8) | 40 | (48.2) |  | 73 | (17.9) | 39 | (53.4) | 34 | (46.6) |  |
| UICC stage | 440/1 |  |  |  |  |  | 0.095 ^b^ | 408/33 |  |  |  |  |  | 0.670 ^b^ |
| IA / IB | 75 | (17.0) | 28 | (37.3) | 47 | (62.7) |  | 68 | (16.7) | 31 | (45.6) | 37 | (54.4) |  |
| IIA / IIB | 96 | (21.8) | 50 | (52.1) | 46 | (47.9) |  | 93 | (22.8) | 50 | (53.8) | 43 | (46.2) |  |
| IIIA / IIIB / IIIC | 186 | (42.3) | 100 | (53.8) | 86 | (46.2) |  | 174 | (42.6) | 84 | (48.3) | 90 | (51.7) |  |
| IV | 83 | (18.9) | 43 | (51.8) | 40 | (48.2) |  | 73 | (17.9) | 39 | (53.4) | 34 | (46.6) |  |
| pR status | 434/5 |  |  |  |  |  | 0.474 ^b^ | 403/38 |  |  |  |  |  | 0.535 ^b^ |
| pR0 | 381 | (87.4) | 194 | (50.9) | 187 | (49.1) |  | 356 | (88.3) | 180 | (50.6) | 176 | (49.4) |  |
| pR1 / pR2 | 55 | (12.6) | 25 | (45.5) | 30 | (54.5) |  | 47 | (11.7) | 21 | (44.7) | 26 | (55.3) |  |
| L category | 421/20 |  |  |  |  |  | 0.119 ^b^ | 390/51 |  |  |  |  |  | 1.000 ^b^ |
| L0 | 203 | (48.2) | 94 | (46.3) | 109 | (53.7) |  | 186 | (47.7) | 92 | (49.5) | 94 | (50.5) |  |
| L1 | 218 | (51.8) | 118 | (54.1) | 100 | (45.9) |  | 204 | (52.3) | 100 | (49.0) | 104 | (51.0) |  |
| V category | 420/21 |  |  |  |  |  | 0.878 ^b^ | 389/52 |  |  |  |  |  | 0.750 ^b^ |
| V0 | 373 | (88.8) | 189 | (50.7) | 184 | (49.3) |  | 345 | (88.7) | 169 | (49.0) | 176 | (51.0) |  |
| V1 | 47 | (11.2) | 23 | (48.9) | 24 | (51.1) |  | 44 | (11.3) | 23 | (52.3) | 21 | (47.7) |  |
| H. pylori status | 374/67 |  |  |  |  |  | 0.388 ^a^ | 351/90 |  |  |  |  |  | 0.308 ^a^ |
| Negative | 317 | (84.8) | 155 | (48.9) | 162 | (51.1) |  | 295 | (84.0) | 144 | (48.8) | 151 | (51.2) |  |
| Positive | 57 | (15.2) | 32 | (56.1) | 25 | (43.9) |  | 56 | (16.0) | 32 | (57.1) | 24 | (42.9) |  |
| HER2 status | 412/29 |  |  |  |  |  | 0.031 ^a *^ | 384/57 |  |  |  |  |  | 0.109 ^a^ |
| Negative | 378 | (91.7) | 199 | (52.6) | 179 | (47.4) |  | 350 | (91.1) | 175 | (50.0) | 175 | (50.0) |  |
| Positive | 34 | (8.3) | 11 | (32.4) | 23 | (67.6) |  | 34 | (8.9) | 12 | (35.3) | 22 | (64.7) |  |
| cMET status | 430/11 |  |  |  |  |  | 1.000 ^a^ | 401/40 |  |  |  |  |  | 0.557 ^a^ |
| Negative | 397 | (92.3) | 198 | (49.9) | 199 | (50.1) |  | 374 | (93.3) | 185 | (49.5) | 189 | (50.5) |  |
| Positive | 33 | (7.7) | 17 | (51.5) | 16 | (48.5) |  | 27 | (6.7) | 15 | (55.6) | 12 | (44.4) |  |
| EBV status | 431/10 |  |  |  |  |  | < 0.001 ^a^ | 400/41 |  |  |  |  |  | < 0.001 ^a^ |
| Negative | 412 | (95.6) | 216 | (52.4) | 196 | (47.6) |  | 383 | (95.8) | 198 | (51.7) | 185 | (48.3) |  |
| Positive | 19 | (4.4) | 0 | (0) | 19 | (100) |  | 17 | (4.2) | 1 | (5.9) | 16 | (94.1) |  |
| MSI status | 429/12 |  |  |  |  |  | 0.003 ^a^ | 398/43 |  |  |  |  |  | 0.005 ^a^ |
| Negative (MSS) | 397 | (92.5) | 209 | (52.6) | 188 | (47.4) |  | 366 | (92.0) | 191 | (52.2) | 175 | (47.8) |  |
| Positive | 32 | (7.5) | 8 | (25.0) | 24 | (75.0) |  | 32 | (8.0) | 8 | (25.0) | 24 | (75.0) |  |
| PD-L1 in tumour cells ^c^ | 419/22 |  |  |  |  |  | < 0.001 ^b^ | 388/53 |  |  |  |  |  | < 0.001 ^b^ |
| Negative (IRS ≤ 2) | 319 | (76.1) | 181 | (56.7) | 138 | (43.3) |  | 292 | (75.3) | 165 | (56.5) | 127 | (43.5) |  |
| Positive (IRS > 2) | 100 | (23.9) | 27 | (27.0) | 73 | (73.0) |  | 96 | (24.7) | 27 | (28.1) | 69 | (71.9) |  |
| PD-L1 in immune cells ^c^ | 419/22 |  |  |  |  |  | 0.015 ^b *^ | 388/53 |  |  |  |  |  | 0.075 ^b^ |
| Negative (QS ≤ 1) | 267 | (63.7) | 145 | (54.3) | 122 | (45.7) |  | 243 | (62.6) | 129 | (53.1) | 114 | (46.9) |  |
| Positive (QS > 1) | 152 | (36.3) | 63 | (41.4) | 89 | (58.6) |  | 145 | (37.4) | 63 | (43.4) | 82 | (56.6) |  |
| PD-1 in immune cells | 422/19 |  |  |  |  |  | < 0.001 ^a^ | 391/50 |  |  |  |  |  | < 0.001 ^a^ |
| Not present | 191 | (45.3) | 119 | (62.3) | 72 | (37.7) |  | 174 | (44.5) | 105 | (60.3) | 69 | (39.7) |  |
| Present | 231 | (54.7) | 91 | (39.4) | 140 | (60.6) |  | 217 | (55.5) | 89 | (41.0) | 128 | (59.0) |  |

^a^ Fisher's exact test, ^b^ Kendall's tau test; ^c^ cut-offs used by Böger et al.; IRS, immunoreactivity score; QS, quantity score; ^*^ statistically non-significant after multiple testing correction.

**Supplement table 4** Neoadjuvant treated GC: Association of LAG3+ cell density (dichotomized by median) with demographical and clinicopathological patient characteristics

|  |  | | **Tumour center** | | | | |  | | **Invasive margin** | | | | |
| --- | --- | --- | --- | --- | --- | --- | --- | --- | --- | --- | --- | --- | --- | --- |
| **Characteristics** | **Valid/missing** | | **LAG3 low** | | **LAG3 high** | | ***p*-value** | **Valid/missing** | | **LAG3 low** | | **LAG3 high** | | ***p*-value** |
|  | **n** | **(%)** | **n** | **(%)** | **n** | **(%)** |  | **n** | **(%)** | **n** | **(%)** | **n** | **(%)** |  |
| Sex | 139/0 |  |  |  |  |  | 0.303 ^a^ | 124/15 |  |  |  |  |  | 0.189 ^a^ |
| Male | 110 | (79.1) | 52 | (47.3) | 58 | (52.7) |  | 98 | (79.0) | 53 | (54.1) | 45 | (45.9) |  |
| Female | 29 | (20.9) | 17 | (58.6) | 12 | (41.4) |  | 26 | (21.0) | 10 | (38.5) | 16 | (61.5) |  |
| Age | 139/0 |  |  |  |  |  | 0.028 ^b *^ | 124/15 |  |  |  |  |  | 0.288 ^b^ |
| < 64 years | 69 | (49.6) | 41 | (59.4) | 28 | (40.6) |  | 59 | (47.6) | 33 | (55.9) | 26 | (44.1) |  |
| ≥ 64 years | 70 | (50.4) | 28 | (40.0) | 42 | (60.0) |  | 65 | (52.4) | 30 | (46.2) | 35 | (53.8) |  |
| Location | 139/0 |  |  |  |  |  | 0.265 ^a^ | 124/15 |  |  |  |  |  | 0.844 ^a^ |
| Proximal stomach | 99 | (71.2) | 46 | (46.5) | 53 | (53.5) |  | 88 | (71.0) | 44 | (50.0) | 44 | (50.0) |  |
| Distal stomach | 40 | (28.8) | 23 | (57.5) | 17 | (42.5) |  | 36 | (29.0) | 19 | (52.8) | 17 | (47.2) |  |
| Laurén phenotype | 139/0 |  |  |  |  |  | 0.066 ^a^ | 124/15 |  |  |  |  |  | 0.195 ^a^ |
| Intestinal | 73 | (52.5) | 33 | (45.2) | 40 | (54.8) |  | 68 | (54.8) | 33 | (48.5) | 35 | (51.5) |  |
| Diffuse | 27 | (19.4) | 19 | (70.4) | 8 | (29.6) |  | 23 | (18.5) | 16 | (69.6) | 7 | (30.4) |  |
| Mixed | 29 | (20.9) | 11 | (37.9) | 18 | (62.1) |  | 24 | (19.4) | 11 | (45.8) | 13 | (54.2) |  |
| Unclassified | 10 | (7.2) | 6 | (60.0) | 4 | (40.0) |  | 9 | (7.3) | 3 | (33.3) | 6 | (66.7) |  |
| ypT category | 139/0 |  |  |  |  |  | 0.048 ^b *^ | 124/15 |  |  |  |  |  | 0.015 ^b *^ |
| pT1 (a/b) | 18 | (13.0) | 6 | (33.3) | 12 | (66.7) |  | 14 | (11.3) | 5 | (35.7) | 9 | (64.3) |  |
| pT2 | 23 | (16.5) | 10 | (43.5) | 13 | (56.5) |  | 20 | (16.1) | 7 | (35.0) | 13 | (65.0) |  |
| pT3 | 88 | (63.3) | 46 | (52.3) | 42 | (47.7) |  | 81 | (65.3) | 44 | (54.3) | 37 | (45.7) |  |
| pT4 (a/b) | 10 | (7.2) | 7 | (70.0) | 3 | (30.0) |  | 9 | (7.3) | 7 | (77.8) | 2 | (22.2) |  |
| ypT category | 139/0 |  |  |  |  |  | 0.137 ^a^ | 124/15 |  |  |  |  |  | 0.044 ^a *^ |
| pT1 (a/b) / pT2 | 41 | (29.5) | 16 | (39.0) | 25 | (61.0) |  | 34 | (27.4) | 12 | (35.3) | 22 | (64.7) |  |
| pT3 / pT4 (a/b) | 98 | (70.5) | 53 | (54.1) | 45 | (45.9) |  | 90 | (72.6) | 51 | (56.7) | 39 | (43.3) |  |
| ypN category | 139/0 |  |  |  |  |  | 0.021 ^b *^ | 124/15 |  |  |  |  |  | 0.025 ^b *^ |
| pN0 | 44 | (31.7) | 20 | (45.5) | 24 | (54.5) |  | 38 | (30.6) | 15 | (39.5) | 23 | (60.5) |  |
| pN1 | 36 | (25.9) | 12 | (33.3) | 24 | (66.7) |  | 32 | (25.8) | 15 | (46.9) | 17 | (53.1) |  |
| pN2 | 34 | (25.2) | 19 | (54.3) | 16 | (45.7) |  | 32 | (25.8) | 18 | (56.3) | 14 | (43.8) |  |
| pN3 (a/b) | 24 | (17.3) | 18 | (75.0) | 6 | (25.0) |  | 22 | (17.7) | 15 | (68.2) | 7 | (31.8) |  |
| ypN category | 139/0 |  |  |  |  |  | 0.585 ^a^ | 124/15 |  |  |  |  |  | 0.120 ^a^ |
| pN0 | 44 | (31.7) | 20 | (45.5) | 24 | (54.5) |  | 38 | (30.6) | 15 | (39.5) | 23 | (60.5) |  |
| pN+ | 95 | (68.1) | 49 | (51.6) | 46 | (48.4) |  | 86 | (69.4) | 48 | (55.8) | 38 | (44.2) |  |
| ypM category | 139/0 |  |  |  |  |  | 0.366 ^b^ | 124/15 |  |  |  |  |  | 0.488 ^b^ |
| M0 | 128 | (92.1) | 62 | (48.4) | 66 | (51.6) |  | 116 | (93.5) | 60 | (51.7) | 56 | (48.3) |  |
| M1 | 11 | (7.9) | 7 | (63.6) | 4 | (36.4) |  | 8 | (6.5) | 3 | (37.5) | 5 | (62.5) |  |
| UICC stage | 139/0 |  |  |  |  |  | 0.300 ^b^ | 124/15 |  |  |  |  |  | 0.170 ^b^ |
| IA / IB | 21 | (15.1) | 8 | (38.1) | 13 | (61.9) |  | 18 | (14.5) | 5 | (27.8) | 13 | (72.2) |  |
| IIA / IIB | 25 | (18.0) | 12 | (48.0) | 13 | (52.0) |  | 22 | (17.7) | 12 | (54.5) | 10 | (45.5) |  |
| IIIA / IIIB / IIIC | 76 | (54.7) | 40 | (52.6) | 36 | (47.4) |  | 69 | (55.7) | 38 | (55.1) | 31 | (44.9) |  |
| IV / IVA / IVB | 17 | (12.2) | 9 | (52.9) | 8 | (47.1) |  | 15 | (12.1) | 8 | (53.3) | 7 | (46.7) |  |
| pR status | 134/5 |  |  |  |  |  | 0.445 ^b^ | 120/19 |  |  |  |  |  | 0.175 ^b^ |
| pR0 | 117 | (87.3) | 56 | (47.9) | 61 | (52.1) |  | 105 | (87.5) | 49 | (46.7) | 56 | (53.3) |  |
| pR1 / pR2 | 17 | (12.7) | 10 | (58.8) | 7 | (41.2) |  | 15 | (12.5) | 10 | (66.7) | 5 | (33.3) |  |
| L category | 136/3 |  |  |  |  |  | 0.856 ^b^ | 121/18 |  |  |  |  |  | 0.709 ^b^ |
| L0 | 90 | (66.2) | 46 | (51.1) | 44 | (48.9) |  | 78 | (64.5) | 41 | (52.6) | 37 | (47.4) |  |
| L1 | 46 | (33.8) | 22 | (47.8) | 24 | (52.2) |  | 43 | (35.5) | 21 | (48.8) | 22 | (51.2) |  |
| V category | 133/6 |  |  |  |  |  | 0.207 ^b^ | 118/21 |  |  |  |  |  | 0.054 ^b^ |
| V0 | 122 | (91.7) | 59 | (48.4) | 63 | (51.6) |  | 107 | (90.7) | 51 | (47.7) | 56 | (52.3) |  |
| V1 | 11 | (8.3) | 8 | (72.7) | 3 | (27.3) |  | 11 | (9.3) | 9 | (81.8) | 2 | (18.2) |  |
| HER2 status | 135/4 |  |  |  |  |  | 0.055 ^a^ | 122/17 |  |  |  |  |  | 1.000 ^a^ |
| Negative | 125 | (92.6) | 59 | (47.2) | 66 | (52.8) |  | 113 | (92.6) | 58 | (51.3) | 55 | (48.7) |  |
| Positive | 10 | (7.4) | 8 | (80.0) | 2 | (20.0) |  | 9 | (7.4) | 5 | (55.6) | 4 | (44.4) |  |
| cMET status | 131/8 |  |  |  |  |  | 0.427 ^a^ | 117/22 |  |  |  |  |  | 1.000 ^a^ |
| Negative | 125 | (95.4) | 59 | (47.2) | 66 | (52.8) |  | 112 | (95.7) | 59 | (52.7) | 53 | (47.3) |  |
| Positive | 6 | (4.6) | 4 | (66.7) | 2 | (33.3) |  | 5 | (4.3) | 3 | (60.0) | 2 | (40.0) |  |
| EBV status | 120/19 |  |  |  |  |  | 0.110 ^a^ | 108/31 |  |  |  |  |  | 0.595 ^a^ |
| Negative | 117 | (97.5) | 62 | (53.0) | 55 | (47.0) |  | 105 | (97.2) | 57 | (54.3) | 48 | (45.7) |  |
| Positive | 3 | (2.5) | 0 | (0.0) | 3 | (100) |  | 3 | (2.8) | 1 | (33.3) | 2 | (66.7) |  |
| MSI status | 127/12 |  |  |  |  |  | 0.713 ^a^ | 115/24 |  |  |  |  |  | 0.708 ^a^ |
| Negative (MSS) | 120 | (94.5) | 62 | (51.7) | 58 | (48.3) |  | 108 | (93.9) | 57 | (52.8) | 51 | (47.2) |  |
| Positive (MSI) | 7 | (5.5) | 3 | (42.9) | 4 | (57.1) |  | 7 | (6.1) | 3 | (42.9) | 4 | (57.1) |  |
| PD-L1 in tumour cells ^c^ | 109/30 |  |  |  |  |  | 0.024 ^b *^ | 100/39 |  |  |  |  |  | 0.005 ^b^ |
| Negative (IRS = 0) | 84 | (77.1) | 49 | (58.3) | 35 | (41.7) |  | 76 | (76.0) | 48 | (63.2) | 28 | (36.8) |  |
| Positive (IRS > 0) | 25 | (22.9) | 8 | (32.0) | 17 | (68.0) |  | 24 | (24.0) | 7 | (29.2) | 17 | (70.8) |  |
| PD-L1 in immune cells ^c^ | 113/26 |  |  |  |  |  | < 0.001 ^b^ | 100/39 |  |  |  |  |  | 0.009 ^b *^ |
| Negative (QS ≤ 1) | 66 | (58.4) | 45 | (68.2) | 21 | (31.8) |  | 55 | (55.0) | 37 | (67.3) | 18 | (32.7) |  |
| Positive (QS > 1) | 47 | (41.6) | 14 | (29.8) | 33 | (70.2) |  | 45 | (45.0) | 18 | (40.0) | 27 | (60.0) |  |
| PD-1 in immune cells | 113/26 |  |  |  |  |  | 0.441 ^a^ | 100/39 |  |  |  |  |  | 0.125 ^a^ |
| Not present | 7 | (6.2) | 5 | (71.4) | 2 | (28.6) |  | 7 | (7.0) | 6 | (85.7) | 1 | (14.3) |  |
| Present | 106 | (93.8) | 54 | (50.9) | 52 | (49.1) |  | 93 | (93.0) | 49 | (52.7) | 44 | (47.3) |  |

^a^ Fisher's exact test, ^b^ Kendall's tau test; ^c^ cut-offs used by Schoop et al.; IRS, immunoreactivity score; QS, quantity score; ^*^ statistically non-significant after multiple testing correction.
